# *Staphylococcus aureus* HemX Modulates Glutamyl-tRNA Reductase Abundance To Regulate Heme Biosynthesis

**DOI:** 10.1128/mBio.02287-17

**Published:** 2018-02-06

**Authors:** Jacob E. Choby, Caroline M. Grunenwald, Arianna I. Celis, Svetlana Y. Gerdes, Jennifer L. DuBois, Eric P. Skaar

**Affiliations:** a Department of Pathology, Microbiology, & Immunology, Vanderbilt University Medical Center, Nashville, Tennessee, USA; b Vanderbilt Institute for Infection, Immunology, and Inflammation, Vanderbilt University Medical Center, Nashville, Tennessee, USA; c Graduate Program in Microbiology & Immunology, Vanderbilt University, Nashville, Tennessee, USA; d Department of Chemistry and Biochemistry, Montana State University, Bozeman, Montana, USA; e Fellowship for Interpretation of Genomes, Burr Ridge, Illinois, USA; Nanyang Technological University

**Keywords:** *Staphylococcus aureus*, heme, tetrapyrroles

## Abstract

*Staphylococcus aureus* is responsible for a significant amount of devastating disease. Its ability to colonize the host and cause infection is supported by a variety of proteins that are dependent on the cofactor heme. Heme is a porphyrin used broadly across kingdoms and is synthesized *de novo* from common cellular precursors and iron. While heme is critical to bacterial physiology, it is also toxic in high concentrations, requiring that organisms encode regulatory processes to control heme homeostasis. In this work, we describe a posttranscriptional regulatory strategy in *S. aureus* heme biosynthesis. The first committed enzyme in the *S. aureus* heme biosynthetic pathway, glutamyl-tRNA reductase (GtrR), is regulated by heme abundance and the integral membrane protein HemX. GtrR abundance increases dramatically in response to heme deficiency, suggesting a mechanism by which *S. aureus* responds to the need to increase heme synthesis. Additionally, HemX is required to maintain low levels of GtrR in heme-proficient cells, and inactivation of *hemX* leads to increased heme synthesis. Excess heme synthesis in a Δ*hemX* mutant activates the staphylococcal heme stress response, suggesting that regulation of heme synthesis is critical to reduce self-imposed heme toxicity. Analysis of diverse organisms indicates that HemX is widely conserved among heme-synthesizing bacteria, suggesting that HemX is a common factor involved in the regulation of GtrR abundance. Together, this work demonstrates that *S. aureus* regulates heme synthesis by modulating GtrR abundance in response to heme deficiency and through the activity of the broadly conserved HemX.

## IMPORTANCE

*Staphylococcus aureus* is a leading cause of skin and soft tissue infections, endocarditis, bacteremia, and osteomyelitis, making it a critical health care concern. Development of new antimicrobials against *S. aureus* requires knowledge of the physiology that supports this organism’s pathogenesis. One component of staphylococcal physiology that contributes to growth and virulence is heme. Heme is a widely utilized cofactor that enables diverse chemical reactions across many enzyme families. *S. aureus* relies on many critical heme-dependent proteins and is sensitive to excess heme toxicity, suggesting *S. aureus* must maintain proper intracellular heme homeostasis. Because *S. aureus* provides heme for heme-dependent enzymes via synthesis from common precursors, we hypothesized that regulation of heme synthesis is one mechanism to maintain heme homeostasis. In this study, we identify that *S. aureus* posttranscriptionally regulates heme synthesis by restraining abundance of the first heme biosynthetic enzyme, GtrR, via heme and the broadly conserved membrane protein HemX.

## INTRODUCTION

The tetrapyrrole cofactor heme is critical to the physiology of organisms from humans to bacteria. Heme is composed of a porphyrin ring complexed to iron at its center, making it an excellent redox-active moiety for a variety of enzymes. Across kingdoms, heme is used to shuttle electrons in the respiratory chain and is also required for the function of many critical proteins, including nitric oxide synthase, catalase, and hemoglobin. To satisfy the cellular need for heme, most heme-dependent organisms synthesize heme *de novo* from simple and abundant precursors.

The versatility of heme as a cofactor is based on its reactivity, which also results in its toxicity at high concentrations. Excess heme can cause damage to cellular macromolecules, and the redox cycling of heme-iron produces reactive oxygen species via Fenton chemistry (1). Bacteria encode a variety of mechanisms to resist heme toxicity (1), but the most important of these strategies may be the prevention of self-imposed toxicity by regulating endogenous heme synthesis. A variety of transcriptional and posttranscriptional strategies have evolved to regulate heme synthesis centered around providing sufficient heme to occupy hemoproteins while preventing excess heme synthesis to limit unnecessary consumption of substrates and preclude toxicity.

In this study, we sought to uncover regulatory pathways controlling heme synthesis in the human pathogen *Staphylococcus aureus*. *S. aureus* is a Gram-positive bacterium that causes a variety of devastating diseases, including skin and soft tissue infections, osteomyelitis, endocarditis, and bacteremia (2). *S. aureus*, as a facultative anaerobe, generates energy through aerobic respiration, anaerobic respiration, or fermentation. The final step in aerobic respiration is reduction of oxygen to water, which *S. aureus* performs with either of the heme-dependent QoxABCD or CydAB terminal oxidases (3, 4). Although a great deal is known about heme synthesis, heme utilization, and heme toxicity in *S. aureus*, no heme synthesis regulatory pathway has been identified in this organism. *S. aureus* encodes the newly appreciated coproporphyrin-dependent heme synthesis pathway to populate its hemoproteins (5, 6). These include the terminal oxidases, catalase, and bacterial nitric oxide synthase, all of which contribute to growth, protection from host defenses, and pathogenesis (3, 7–9). Under conditions of excess exogenous heme, the heme stress response in *S. aureus* is activated by the heme-sensing two-component system HssRS, which regulates the transcription of a putative efflux pump, HrtAB. This system is critical for growth and survival in toxic concentrations of heme and modulates pathogenesis in a murine model of disease (10). Sensing or regulatory pathways that connect heme synthesis with heme availability, hemoprotein abundance, or HssRS activation have not been identified.

*S. aureus* synthesizes δ-aminolevulinic acid (ALA), the first dedicated and universal precursor for protoheme synthesis, via the conversion of glutamyl-tRNA to glutamate-1-semialdehyde by glutamyl-tRNA reductase (GtrR) and subsequent production of ALA by glutamate-1-semialdehyde 2,1-aminomutase (11–13). Uroporphyrinogen is the precursor to heme, siroheme, and other tetrapyrroles, and the stepwise transformation of ALA to uroporphyrinogen comprises the core of the synthesis pathway. The pathway from uroporphyrinogen to heme was historically considered to be universally conserved for all organisms. However, the field’s understanding of bacterial heme synthesis has undergone a revolution as recent studies uncovered diversity in bacterial strategies to convert uroporphyrinogen to heme (reviewed in reference 14). Gram-positive bacteria proceed through a coproporphyrin-dependent branch (5, 6, 15) that is unique from the classic protoporphyrin-dependent branch in humans and many Gram-negative model organisms.

In this work, we identify GtrR abundance as a critical regulator of *S. aureus* heme synthesis. GtrR is posttranscriptionally maintained at low abundance in heme-proficient cells by the membrane protein HemX, but levels increase when *S. aureus* is deprived of heme. Without HemX regulation, GtrR abundance increases, which results in the concomitant increase in flux through the heme synthesis pathway and accumulation of heme. This excess heme synthesis activates HssRS and disrupts iron homeostasis. Together, this report reveals that *S. aureus* regulates heme synthesis by modulating GtrR abundance via intracellular heme levels and the widely conserved HemX.

## RESULTS

### GtrR abundance increases specifically in response to heme deficiency.

**FIG 1:**
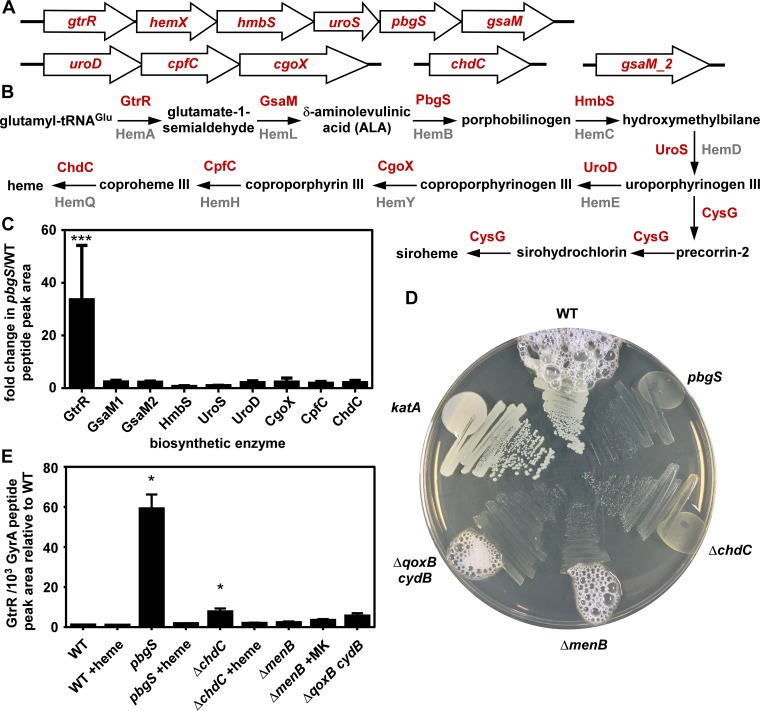
Heme deficiency increases GtrR abundance. (A) The genes encoding heme biosynthesis enzymes are located at four chromosomal loci. (B) An overview of the *S. aureus* heme and siroheme biosynthetic pathway. In red are the updated enzyme names set forth by Dailey and colleagues (14), which correspond to the previously used gene locus names in gray. (C) The abundance of each biosynthetic enzyme was measured by LC-MRM-MS/MS and quantified by integrated chromatogram peak areas. Graphed is the ratio of each enzyme’s abundance in a strain lacking *pbgS* relative to WT *S. aureus*; the data are the average from a single experiment performed in biological triplicate with standard deviation shown. Statistical significance was determined using a one-way analysis of variance (ANOVA) with Dunnett’s correction for multiple comparisons, using a reference value of 1.0. ***, *P* < 0.001. (D) The *S. aureus* strains listed were streaked onto rich agar medium plates, and after growth, hydrogen peroxide was added at the perimeter of each streak. (E) The abundance of GtrR was measured by LC-MRM-MS/MS in *S. aureus* strains treated with vehicle, heme, or menaquinone (MK). The data are the average from a single experiment performed in biological triplicate with standard deviation shown. Statistical significance was determined using a one-way ANOVA with Dunnett’s correction for multiple comparisons, comparing GtrR abundance for each condition relative to the WT. *, *P* < 0.05.

To identify key steps in the regulation of heme synthesis (Fig. 1A and B), we measured the abundance of each biosynthetic enzyme by liquid chromatography-multiple reaction monitoring-tandem mass spectrometry (LC-MRM-MS/MS). This technique allows for quantification with high resolution of even very-low-abundance cellular proteins (16). We hypothesized that comparing the *S. aureus* wild type (WT) to a strain incapable of synthesizing heme (*pbgS* mutant) (Fig. 1A and B) would allow the identification of specific steps in heme synthesis that respond to cellular heme content, directly or indirectly. Abundance of GtrR is approximately 30-fold higher in the *pbgS* mutant relative to the WT, while the abundances of all other biosynthetic enzymes are nearly unchanged (Fig. 1C). In WT cells, GtrR abundance is low relative to other heme synthesis enzymes (see Fig. S1 in the supplemental material). The *pbgS* mutant is a heme auxotroph and therefore adopts the respiration-deficient small-colony variant (SCV) phenotype. SCVs arise as the result of inactivation of respiration via inactivation of heme synthesis, the terminal oxidases, or the electron carrier menaquinone, and SCVs have a dramatically different physiology than respiration-proficient cells (17). Therefore, we sought to determine whether the increase in GtrR abundance in the *pbgS* mutant was the result of heme deficiency or a general defect in respiration. To confirm that the menaquinone auxotroph SCV Δ*menB* strain and the Δ*qoxB cydB* strain lacking both terminal cytochrome oxidases synthesize heme despite being unable to respire, each strain was streaked onto agar and assessed for catalase activity (Fig. 1D). Activity of the heme-dependent catalase KatA leads to the production of oxygen bubbles when hydrogen peroxide is added. The Δ*menB* and Δ*qoxB cydB* mutants produce bubbles, demonstrating that these SCVs synthesize heme and are not heme auxotrophs. We measured GtrR abundance in a variety of SCV strains by LC-MRM-MS/MS. GtrR abundance increases relative to the WT only in *pbgS* and Δ*chdC* strains (Fig. 1B and E), which are heme auxotroph SCVs (Fig. 1D). When chemically complemented with heme, GtrR abundance returned to WT levels for both strains. GtrR levels do not increase in the Δ*menB* or Δ*qoxB cydB* strain. Together these data demonstrate that the abundance of GtrR is low in heme-proficient cells but increases specifically in response to heme deficiency.

10.1128/mBio.02287-17.2 FIG S1  GtrR abundance is uniquely low among heme synthesis enzymes. Shown is the abundance of each heme synthesis enzyme as measured by LC-MRM-MS/MS in WT cells. The data are the average from a single experiment performed in biological triplicate with standard deviation shown. Download FIG S1, TIF file, 0.9 MB. Copyright © 2018 Choby et al. 2018 Choby et al. This content is distributed under the terms of the Creative Commons Attribution 4.0 International license.

### HemX controls GtrR abundance in heme-proficient cells to regulate heme synthesis.

**FIG 2:**
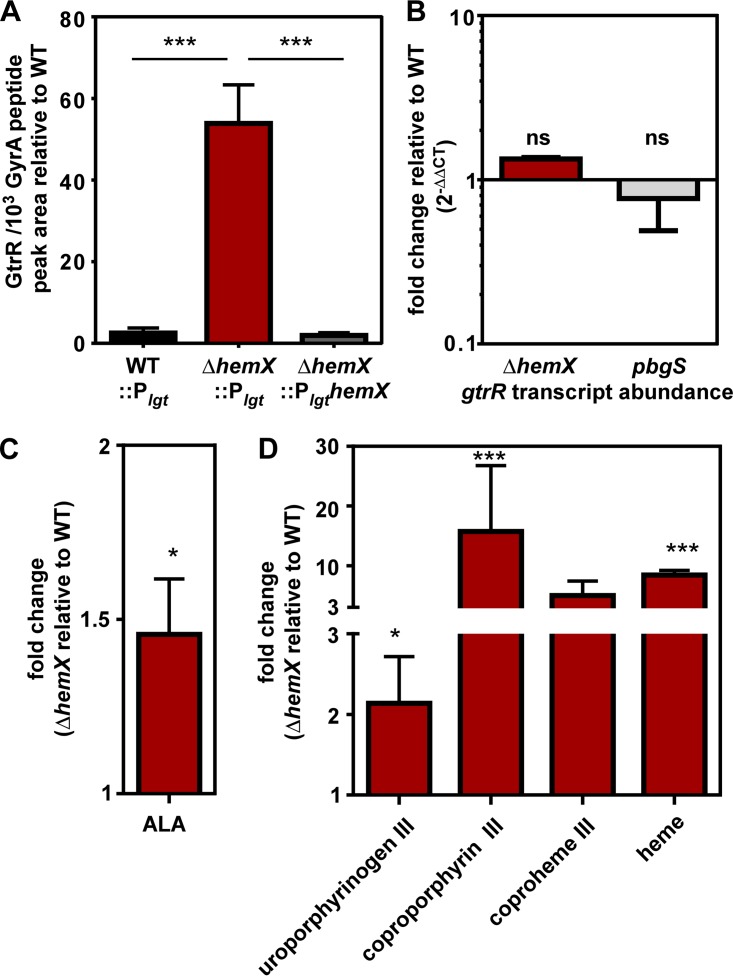
HemX regulates heme synthesis by maintaining low levels of GtrR in heme-proficient cells. (A) The abundance of GtrR was measured by LC-MRM-MS/MS in multiple *S. aureus* strains. The data are the average from a single experiment performed in biological triplicate with standard deviation shown. Statistical significance was determined using a one-way ANOVA with Dunnett’s correction for multiple comparisons, comparing GtrR abundance for each strain relative to the Δ*hemX*::P_*lgt*_ strain. ***, *P* < 0.001. (B) Steady-state transcript abundance of *gtrR* mRNA isolated from mid-exponential growth of *S. aureus* strains was measured by qRT-PCR and is graphed as fold change relative to the WT. Data are combined from two independent experiments in biological triplicate with standard deviation shown. “ns” indicates no significance by one-way ANOVA with Dunnett’s correction for multiple comparisons, comparing fold change of the *pbgS* and Δ*hemX* strains to the WT. (C) δ-Aminolevulinic acid (ALA) abundance was measured in *S. aureus* strains by a spectrophotometric quantification. Graphed is the fold change of ALA in the Δ*hemX* mutant relative to the WT, with data combined from two independent experiments with three biological replicates with standard error of the mean shown. (D) Uroporphyrinogen III (detected as uroporphyin III), coproporphyrin III, coproheme III, and heme were quantified by LC-qTOF-MS. Graphed is the fold change of metabolite abundance in the Δ*hemX* mutant relative to the WT, from a single experiment performed in biological triplicate with standard error of the mean shown. For panels C and D, statistical significance was determined with Student’s *t* test comparing the Δ*hemX* mutant to the WT before data transformation to fold change. *, *P* < 0.05; ***, *P* < 0.0001.

Among both Gram-negative and Gram-positive bacteria, regulation of GtrR abundance is a common feature of heme synthesis regulation pathways (18–20). In the model organism *Bacillus subtilis*, which is also a member of the *Firmicutes* phylum, GtrR abundance is impacted by the membrane protein HemX through an unknown mechanism (18, 21). While *S. aureus* is in the same *Bacillales* order as *B. subtilis*, *S. aureus* heme homeostasis is distinct because of its access to host heme and its resistance to heme toxicity mediated by HssRS. Both *B. subtilis* and *S. aureus* carry an operon comprised of *gtrR-hemX-hmbS-uroS-pgbS-gsaM* (formerly *hemAXCBDL*) (22, 23). We therefore hypothesized that in *S. aureus*, HemX also impacts GtrR abundance in heme-proficient cells. We created an in-frame unmarked deletion of *hemX* and integrated either pJC1111 P_*lgt*_ or P*_lgt_hemX* at a neutral site in the chromosome (24). GtrR abundance was measured by LC-MRM-MS/MS and is increased in the Δ*hemX*::P_*lgt*_ strain relative to WT::P_*lgt*_ (Fig. 2A). The phenotype can be complemented when *hemX* is provided in *cis*, showing that it is the result of deletion of *hemX* and not other effects of disrupting the operon. These data are consistent with the hypothesis that HemX regulates GtrR abundance in heme-proficient cells (18, 21).

We next sought to determine whether the increase in GtrR at the protein level in both the *pbgS* and Δ*hemX* strains is the result of an increase in mRNA transcript abundance of *gtrR*. Therefore, the *pbgS* and Δ*hemX* strains were grown to the mid-exponential phase, and RNA was isolated, converted to cDNA, and quantified by quantitative PCR (qPCR) (Fig. 2B). The steady-state mRNA abundance of *gtrR* transcript is unchanged in the Δ*hemX* or *pbgS* strain relative to the WT, suggesting that the increase in GtrR abundance in these strains is not the result of a transcriptional change. Additionally, the increase in GtrR levels in the *pbgS* strain is not affected by the insertion of *ermB* to interrupt the *pbgS* gene, which is upstream of *gsaM* in the operon; there is no change in the transcript abundance of *gsaM* in the *pbgS* strain relative to the WT (see Fig. S2 in the supplemental material).

10.1128/mBio.02287-17.3 FIG S2  The *pbgS* allele is not polar on *gsaM* transcription. Steady-state transcript abundance of *gsaM* mRNA isolated from mid-exponential growth of *S. aureus* strains was measured by qRT-PCR and is graphed as fold change relative to the WT. Data are combined from two independent experiments in biological triplicate with standard deviation shown. “ns” indicates no significance by one-way ANOVA with Dunnett’s correction for multiple comparisons, comparing fold change of the *pbgS* mutant to the WT. Download FIG S2, TIF file, 0.3 MB. Copyright © 2018 Choby et al. 2018 Choby et al. This content is distributed under the terms of the Creative Commons Attribution 4.0 International license.

We hypothesized that the increase in GtrR observed in the Δ*hemX* strain would increase the amount of heme synthesized by increasing abundance of the heme precursors downstream of GtrR. As glutamate-1-semialdehyde is unstable and can convert to δ-aminolevulinic acid (ALA) in the absence of enzyme (25), we measured ALA abundance via a colorimetric method. ALA abundance increases approximately 50% in the Δ*hemX* strain relative to the WT (Fig. 2C). We subsequently sought to determine the impact of increased ALA availability on downstream heme intermediates and heme abundance. Total cellular porphryins were extracted from the WT and Δ*hemX* strains and analyzed by quantitative exact-mass liquid chromatography-quadrupole time of flight mass spectrometry (LC-qTOF-MS); total extracted ion chromatograms for porphyrins that were observed above the limits of detection are shown in Fig. S3 in the supplemental material, where a dramatic change in porphyrin levels is visible. Based on standard curves for individual porphyrins (including porphobilinogen, uroporphyrins I and III, coproporphyrins I and III, coproheme III, protoporphyrin IX, and heme *b*) and enumeration of colony-forming units, absolute quantifications were obtained and referenced per cell for each porphyrin molecule; data are presented in Fig. 2D in terms of fold change relative to the WT. As shown in Fig. 2D; the Δ*hemX* strain exhibits increased abundance of uroporphyrin III, coproporphyrin III, coproheme III, and heme *b* relative to the WT. Notably, because samples were prepared aerobically, the metabolite uroporphyrinogen III was detected as uroporphyrin III, in which its methylene bridge carbons have spontaneously oxidized in air; likewise, any coproporphyrinogen III that might have been present would be detected as the oxidation product, coproporphyrin III, which is also the product of the enzyme CgoX (Fig. 1). Hydroxymethylbilane spontaneously cyclizes to uroporphyrinogen I, which is decarboxylated by UroD to coproporphyrinogen I. The absence of uroporphyrin or copropophyrin I isomers indicates that hydroxymethylbilane did not accrue in the Δ*hemX* mutant. We hypothesize that, in the presence of excess GtrR, the initial step of the pathway may no longer be rate limiting. This may allow other subsequent steps in the pathway to become partly rate limiting, leading to the observed pattern of metabolite accumulation. Finally, the increase in heme abundance in the Δ*hemX* strain observed by LC-qTOF-MS is complemented when *hemX* is provided in *cis* from a neutral site in the chromosome, as measured by the pyridine hemochromagen method (see Fig. S4A in the supplemental material). Together, these data demonstrate that inactivation of *hemX* results in increased GtrR abundance, which increases abundances of both early- and late-pathway biosynthetic precursors and cellular heme. Therefore, dysregulation of GtrR alone is sufficient to disrupt heme homeostasis.

10.1128/mBio.02287-17.4 FIG S3  Representative extracted ion chromatograms of extracted porphyrins of (A) the *S. aureus* WT and (B) the Δ*hemX* mutant; each is quantified and shown in Fig. 2. Chromatograms for porphyrins above the limits of detection (250 nM) are shown. Download FIG S3, TIF file, 2.7 MB. Copyright © 2018 Choby et al. 2018 Choby et al. This content is distributed under the terms of the Creative Commons Attribution 4.0 International license.

10.1128/mBio.02287-17.5 FIG S4  Excess heme and resistance to heme toxicity in the Δ*hemX* mutant can be complemented. (A) Heme abundance was quantified using a pyridine hemochromagen assay in *S. aureus* strains. Data are combined from three independent experiments with four biological replicates with standard error of the mean shown. Statistical significance was determined by a one-way ANOVA with Tukey’s correction for multiple comparisons, comparing each strain against the others. **, *P* < 0.005. (B) Growth as measured by optical density (600 nm) was monitored over time for *S. aureus* strains in medium containing 0 or 10 µM heme. Strains were grown overnight to the stationary phase in medium alone before inoculation of the growth curve. The data are the average of the means from three independent experiments each in biological triplicate with standard error of the mean shown. (C) Growth as measured by optical density (600 nm) was monitored over time for *S. aureus* strains in medium containing chloramphenicol and 0 or 12.5 µM heme. Strains were grown overnight to stationary phase in medium alone before inoculation of the growth curve. The data are means from three biological replicates with standard error of the mean shown from a single experiment, representative of at least three independent experiments. Download FIG S4, TIF file, 6.8 MB. Copyright © 2018 Choby et al. 2018 Choby et al. This content is distributed under the terms of the Creative Commons Attribution 4.0 International license.

### Excess endogenous heme synthesis in the Δ*hemX* mutant activates the heme stress response.

**FIG 3:**
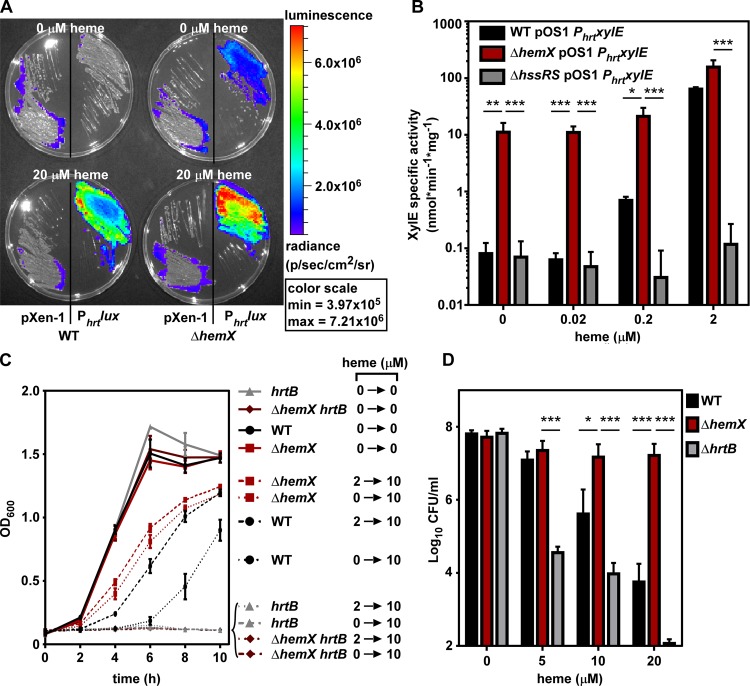
Excess heme synthesis in the Δ*hemX* mutant activates the heme stress response. (A) Bioluminescence was imaged on agar medium plates containing vehicle or heme onto which strains were streaked. All four plates were imaged simultaneously, and luminescence was converted to a heat map with the scale shown on the right. (B) XylE catechol oxidase activity was measured in *S. aureus* strains after growth in vehicle or increasing concentrations of heme. The data are the average from three independent experiments each in biological triplicate with standard deviation shown. Statistical significance was determined using a two-way ANOVA with Tukey’s correction for multiple comparisons, comparing log-transformed data for the Δ*hemX* pOS1 P*_hrt_xylE* strain at each heme concentration to that of each other strain. *, *P* < 0.01; **, *P* < 0.001; ***, *P* < 0.0001. (C) Growth as measured by OD_600_ was monitored over time for *S. aureus* strains in medium containing either vehicle or 10 µM heme. Prior to the measured growth, the strains were pregrown to the stationary phase in medium containing vehicle or 2 µM heme. The data are the average of the means from at least three independent experiments each in biological triplicate with standard error of the mean shown. (D) Viable bacteria from *S. aureus* strains were enumerated after incubation for 2 h in medium containing vehicle or increasing amounts of heme. The data are the average of the means from three independent experiments each in biological triplicate with standard error of the mean shown. The *y* axis is set to the limit of detection. Statistical significance was determined using a two-way ANOVA with Tukey’s correction for multiple comparisons, comparing log-transformed data for the WT and Δ*hrtB* strains to the Δ*hemX* mutant at each heme concentration. *, *P* < 0.01; ***, *P* < 0.0001.

The unregulated GtrR abundance in the Δ*hemX* mutant results in greater cellular heme levels (Fig. 2D), which we hypothesized would activate the heme sensor system HssRS in the absence of exogenous heme, leading to transcriptional induction of the *hrtAB* efflux pump. The WT and Δ*hemX* mutant strains were transformed with plasmids containing the luminescence-producing operon *luxABCDE* cloned from *Photorhabdus luminescens* without a promoter (pXen-1) or controlled by the HssRS-regulated promoter P_*hrt*_. P_*hrt*_ promoter activity, visualized by luminescent imaging, shows that HssRS is activated in the Δ*hemX* strain in the absence of exogenous heme, whereas HssRS is not activated in the WT (Fig. 3A). The P*_hrt_lux* activity in WT becomes apparent when 20 µM exogenous heme is added to the agar medium, and luminescence depends on the heme-responsive P_*hrt*_. To more quantitatively measure P_*hrt*_ activity as a readout of HssRS activation by endogenous heme, we transformed WT, Δ*hemX*, and Δ*hssRS* strains with the pOS1 P*_hrt_xylE* plasmid. These strains report P_*hrt*_ activity with the production of the XylE catechol oxidase enzyme, which can be quantified spectrophotometrically from cell lysate. Data in Fig. 3B demonstrate that in the absence of exogenous heme, P_*hrt*_ is induced in the Δ*hemX* strain. P_*hrt*_ activity does increase in the WT and Δ*hemX* strains in a dose-dependent manner as exogenous heme is added, but P_*hrt*_ activity remains higher in the Δ*hemX* mutant than the WT at all tested heme concentrations. Additionally, XylE activity in this system is dependent on the HssRS two-component system. Taken together, these data suggest that excess endogenous heme synthesized in the Δ*hemX* strain is sufficient to activate the HssRS two-component system.

We next hypothesized that the intermediate levels of HssRS activation in the Δ*hemX* mutant, in the absence of exogenous heme (Fig. 3A and B), would be sufficient to preadapt the Δ*hemX* mutant to heme toxicity. As the HssRS-HrtAB heme stress response provides resistance to heme toxicity, pretreatment with subtoxic concentrations of heme adapts *S. aureus* to subsequent growth in toxic concentrations of heme by activating HssRS and increasing the abundance of HrtAB (10). The WT grown in 10 µM heme without preadaptation has a severe growth defect evident by a 6-h lag time (Fig. 3C). When preadapted in 2 µM heme, the WT demonstrates a reduced lag time and greater overall growth, albeit at a lower rate and yield than when grown without heme. In contrast, the Δ*hemX* mutant grown in 10 µM heme with or without preadaptation exhibits increased growth compared to the WT. The enhanced growth of the Δ*hemX* mutant in 10 µM heme is dependent on the HrtAB efflux system, as the Δ*hemX hrtB* strain does not grow in 10 µM heme (Fig. 3C). Additionally, preadaptation of the Δ*hemX* strain in this assay can be complemented by providing *hemX* in the chromosome (Fig. S4B). Similarly, the Δ*gtrR-hemX* pOS1 P*_lgt_gtrR* strain is resistant to heme toxicity, but becomes sensitive again when *hemX* is introduced on the plasmid (Fig. S4C). This is further evidence that HemX control of GtrR is not transcriptional, as HemX exerts its effect independent of the native *gtrR* promoter and ribosome binding site in this assay. Further, the Δ*hemX* mutant is resistant to the bactericidal effects of acute heme toxicity, compared to a 4-log reduction in viable WT cells after 2 h in the presence of 20 µM heme (Fig. 3D). In sum, these data demonstrate that increased cellular heme in the Δ*hemX* mutant is sufficient to activate HssRS and cause expression of HrtAB, which leads to resistance to heme toxicity.

### Excess heme synthesis disrupts iron homeostasis.

**FIG 4:**
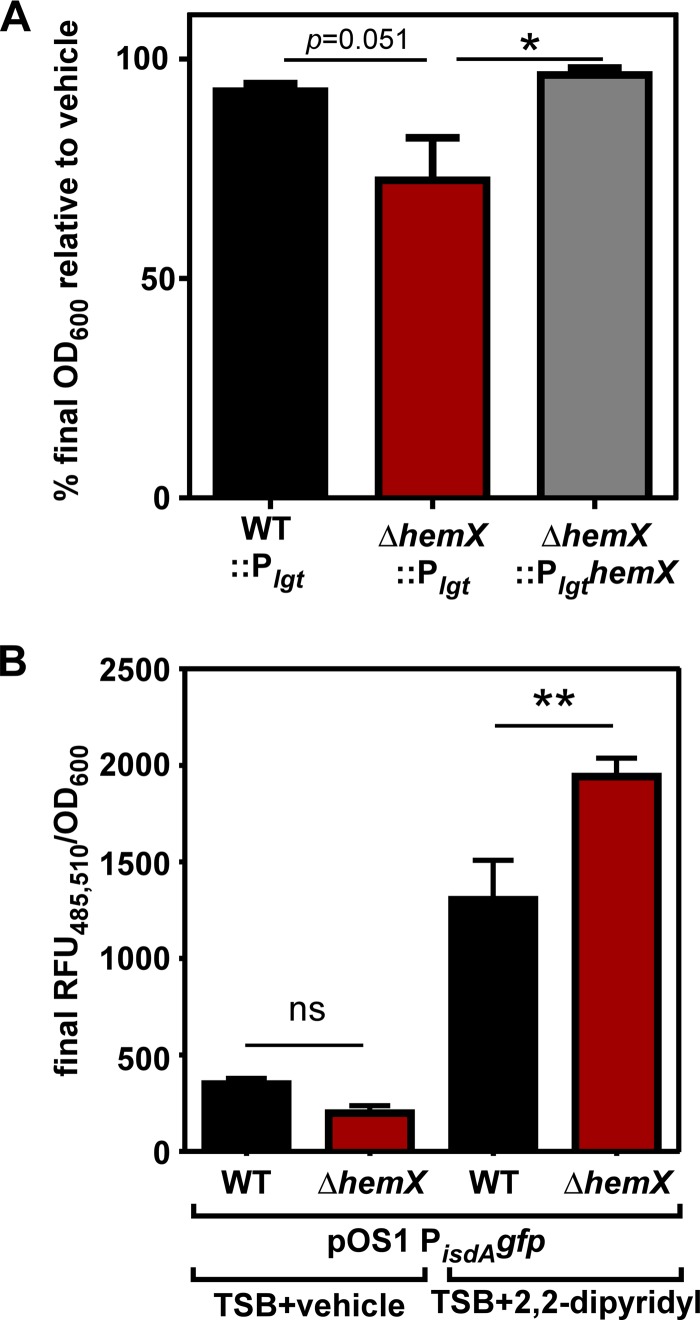
Unregulated heme synthesis alters iron homeostasis. (A) Growth was measured in minimal medium containing vehicle or 1 µM iron chelator EDDHA. Graphed is the final growth as measured by the OD_600_ for each *S. aureus* strain in medium containing EDDHA relative to vehicle. The data are the average of the means from five independent experiments each in at least biological triplicate with standard error of the mean shown. Statistical significance was determined using a one-way ANOVA with Dunnett’s correction for multiple comparisons, comparing the Δ*hemX*::P_*lgt*_ strain to each other strain. *, *P* < 0.05. (B) The activity of the iron limitation-responsive promoter P_*isdA*_ was measured by recording fluorescence intensity over time in rich medium containing vehicle or the iron chelator 2,2-dipyridyl. The data are the average of the means from three independent experiments each in biological triplicate with standard error of the mean shown. Statistical significance was determined using a one-way ANOVA with Sidak’s correction for multiple comparisons, comparing data for the WT and Δ*hemX* mutant under each condition. **, *P* < 0.01; ns, not significant.

Considering that every molecule of heme contains an atom of iron, we hypothesized that unregulated heme synthesis in the Δ*hemX* mutant would consume high levels of iron and alter iron homeostasis. To test this hypothesis, growth in minimal medium containing the iron chelator EDDHA [ethylenediamine-*N*,*N*′-bis(2-hydroxyphenylacetic acid)] was compared to growth in minimal medium alone. As shown in Fig. 4A, the Δ*hemX*::P_*lgt*_ strain demonstrates reduced total yield after growth for 24 h relative to WT::P_*lgt*_ and the complemented Δ*hemX*::P*_lgt_hemX* strain. To corroborate this finding, we assessed promoter activity using a P*_isdA_gfp* reporter plasmid. P_*isdA*_ is controlled by the ferric uptake regulator (Fur) and is derepressed under iron-depleted conditions (26). Data in Fig. 4B show that after growth in rich medium with an alternative iron chelator, 2,2-dipyridyl, the Δ*hemX* pOS1 P*_isdA_gfp* strain has enhanced P_*isdA*_ activity relative to WT pOS1 P*_isdA_gfp*. These data suggest excess heme synthesis depletes the cell of available iron.

### Inactivation of HemX reduces GtrR abundance in heme deficiency.

**FIG 5:**
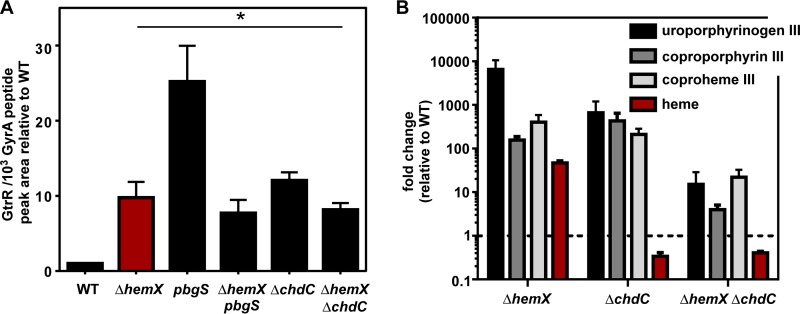
Inactivation of HemX reduces GtrR abundance in heme-deficient strains. (A) The abundance of GtrR was measured by LC-MRM-MS/MS in multiple *S. aureus* strains. The data are the average from a single experiment performed in biological triplicate with standard deviation shown. Statistical significance was determined using a one-way ANOVA with Dunnett’s correction for multiple comparisons, comparing GtrR abundance for each strain relative to WT. *, *P* < 0.05. (B) Uroporphyrinogen III (detected as uroporphyin III), coproporphyrin III, coproheme III, and heme were quantified by LC-qTOF-MS. Graphed is the fold change of metabolite abundance in each mutant relative to the WT from a single experiment performed in biological triplicate with standard error of the mean shown.

Based on the observations that HemX and cellular heme both impact GtrR abundance, we hypothesized that measuring GtrR abundance in a strain lacking *hemX* and unable to synthesize heme would uncover the nature of the relationship between HemX, heme, and GtrR. Surprisingly, GtrR abundance in the Δ*hemX pbgS* strain is unchanged from that in the Δ*hemX* strain and lower than that in the *pbgS* strain (Fig. 5A), and this effect is not the result of a change in *gtrR* transcription in the Δ*hemX pbgS* strain relative to the *pbgS* strain (see Fig. S5 in the supplemental material). Similarly, the Δ*hemX* Δ*chdC* mutant has lower levels of GtrR than the Δ*chdC* mutant (Fig. 5A). To corroborate these findings, we measured total cellular porphyrins by LC-qTOF-MS as in Fig. 2; total extracted ion chromatograms are shown in Fig. S6 in the supplemental material. Consistent with the abundance of GtrR, porphyrin intermediates are drastically increased in the Δ*hemX* mutant relative to the WT. The Δ*chdC* mutant demonstrates intermediate buildup through coproheme because of elevated GtrR levels but is unable to convert coproheme to heme (Fig. 1A). As expected, based on the reduced GtrR abundance shown in Fig. 5A, the porphyin intermediates are at lower levels in the Δ*hemX* Δ*chdC* mutant relative to the Δ*hemX* or Δ*chdC* mutant. These data suggest that heme and HemX do not independently and directly repress GtrR levels, because if so, removal of both would likely have an additive effect on GtrR abundance. Instead the relationship between HemX, heme synthesis, and GtrR levels is still unclear. However, the data are consistent with a model whereby the increase in GtrR levels in heme-deficient strains is dependent on the activity of HemX.

10.1128/mBio.02287-17.6 FIG S5  *gtrR* transcription is unchanged in the Δ*hemX* and Δ*hemX pbgS* strains compared to the *pbgS* strain. Steady-state transcript abundance of *gtrR* mRNA isolated from mid-exponential growth of *S. aureus* strains was measured by qRT-PCR and is graphed as fold change relative to the WT. Data are combined from two independent experiments in biological triplicate with standard deviation shown. “ns” indicates no significance by one-way ANOVA with Dunnett’s correction for multiple comparisons, comparing fold change of the Δ*hemX* mutant and Δ*hemX pbgS* strains to the *pbgS* strain. Download FIG S5, TIF file, 0.7 MB. Copyright © 2018 Choby et al. 2018 Choby et al. This content is distributed under the terms of the Creative Commons Attribution 4.0 International license.

10.1128/mBio.02287-17.7 FIG S6  Representative extracted ion chromatograms of extracted porphyrins of (A) the *S. aureus* WT and (B) the Δ*hemX*, (C) Δ*chdC*, and (D) Δ*hemX* Δ*chdC* strains. Each is quantified and shown in Fig. 5. Chromatograms for porphyrins above the limits of detection (250 nM) are shown. Download FIG S6, TIF file, 3.1 MB. Copyright © 2018 Choby et al. 2018 Choby et al. This content is distributed under the terms of the Creative Commons Attribution 4.0 International license.

### Siroheme synthesis impacts GtrR levels under conditions of nitrite reduction.

**FIG 6:**
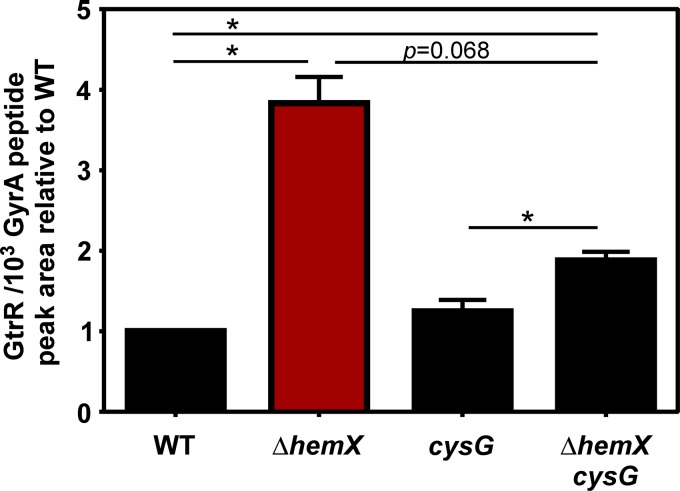
Siroheme synthesis impacts GtrR levels under conditions of nitrite utilization. GtrR was measured by LC-MRM-MS/MS in multiple *S. aureus* strains grown anaerobically with NO_3_ provided as the terminal electron acceptor. The data are the average from a single experiment performed in biological triplicate with standard deviation shown. Statistical significance was determined using a one-way ANOVA with Dunnett’s correction for multiple comparisons, comparing GtrR abundance for each strain relative to the WT. *, *P* < 0.05.

The increase in GtrR levels identified in the Δ*hemX* mutant likely impacts siroheme synthesis, as the cofactor siroheme is synthesized in *S. aureus* from the shared uroporphyrinogen III intermediate (14) (Fig. 1A). We therefore hypothesized that siroheme levels might also affect GtrR abundance. In the experiments presented thus far, in which *S. aureus* is grown aerobically, siroheme has likely not been synthesized. The siroheme synthesis and siroheme-dependent nitrite reductase genes are transcribed primarily under anaerobic conditions (27). To therefore test the role of siroheme, we first identified conditions under which siroheme synthesis via CysG and siroheme-dependent nitrite reduction by the NirD nitrite reductase were important for growth. As demonstrated in Fig. S7 in the supplemental material, when grown anaerobically, the growth of WT is enhanced when the terminal electron acceptor nitrate is provided. Mutants lacking *cysG* or *nirD* cannot grow to WT levels when nitrate is provided, suggesting that WT cells synthesize siroheme and utilize it in NirD. It is thought that under these conditions, the anaerobic nitrate reductase will reduce nitrate to nitrite, followed by NirD-dependent reduction of nitrite. Deletion of *hemX* does not overtly impact nitrite reduction, as the Δ*hemX* strain grows well in nitrate, the Δ*hemX nirD* strain phenocopies the *nirD* strain, and the Δ*hemX cysG* strain phenocopies the *cysG* strain. Therefore, GtrR abundance was measured by LC-MRM-MS/MS after growth in tryptic soy broth (TSB) containing nitrate (Fig. 6). The strain lacking *cysG*, which can make heme but not siroheme, does not demonstrate elevated GtrR levels. However, the Δ*hemX cysG* strain has reduced levels compared to the Δ*hemX* strain, suggesting that siroheme synthesis could impact GtrR regulation.

10.1128/mBio.02287-17.8 FIG S7  Nitrite reductase and the cofactor siroheme are required for full growth with nitrate as an alternative terminal electron acceptor. Shown is growth anaerobically as measured by optical density (600 nm) monitored over time for *S. aureus* strains in medium containing 0 or 40 mM NO_3_. Strains were grown overnight to the stationary phase in medium alone before inoculation of the growth curve. The data are means from three independent experiments with at least three biological replicates. Download FIG S7, TIF file, 4.1 MB. Copyright © 2018 Choby et al. 2018 Choby et al. This content is distributed under the terms of the Creative Commons Attribution 4.0 International license.

### HemX co-occurs with capacity for heme biosynthesis, and the corresponding genes often colocalize on the chromosome.

**FIG 7:**
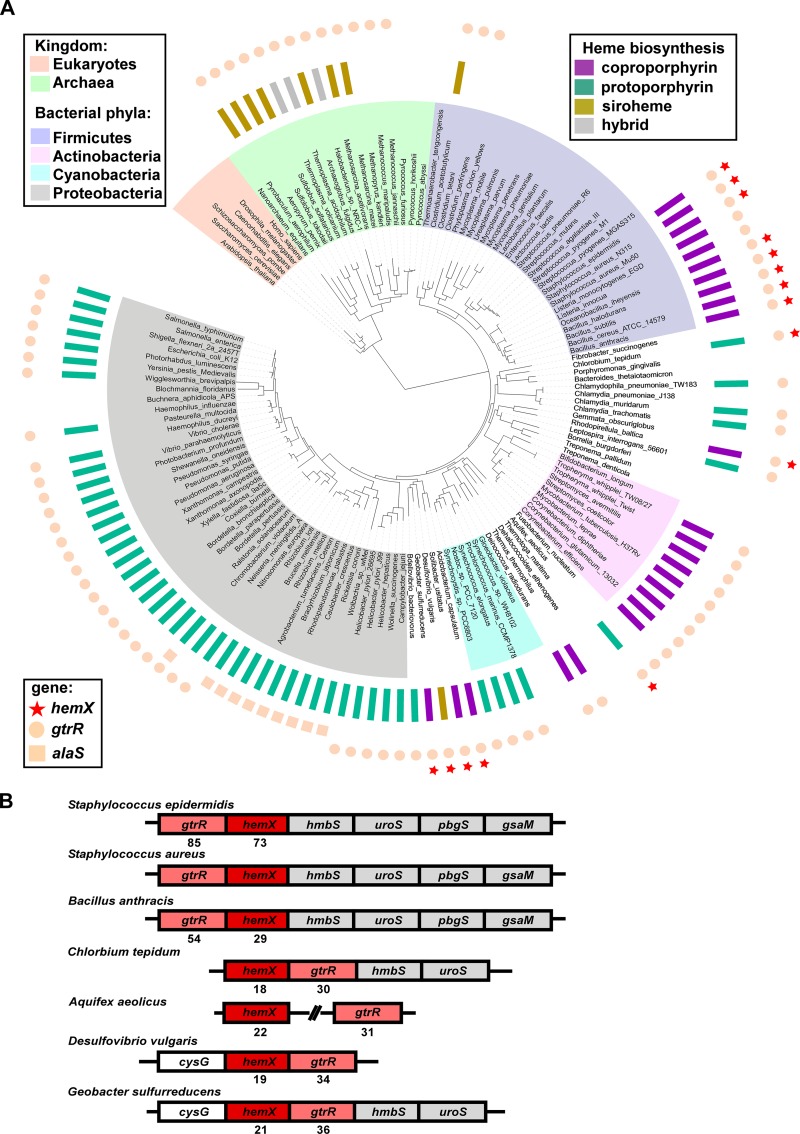
*hemX* is conserved across bacterial phyla and invariably co-occurs (A) and colocalizes (B) with *gtrR*. (A) The occurrence of *hemX* (stars), *gtrR* (circles), and *alaS* (squares) homologues (outermost rings) was mapped onto the tree of life (53). The pathway by which protoheme is synthesized in each of the analyzed organisms is presented in the middle ring as follows (adapted from reference 14): the classic protoporphyrin-dependent pathway (teal), coproporphyrin-dependent path (purple), or siroheme-dependent path (gold). Gray rectangles mark the organisms that contain unusual combinations of genes normally involved in different pathways for protoheme synthesis (hybrid paths [14]). The absence of a rectangle in the middle ring indicates the absence of any known route for protoheme synthesis in an organism. Likewise, the absence of a circle (*gtrR*) or square (*alaS*) in the outermost ring shows the inability of an organism to produce tetrapyrroles of any kind. Note that *hemX* does not occur in such organisms. (B) The immediate genomic neighborhood of the *hemX* gene in seven representative genomes, with ClustalW alignment scores for HemX and GtrR for each organism relative to *S. aureus*.

We hypothesized that *B. subtilis* HemX and *S. aureus* HemX might represent only a subset of HemX homologues that exist across bacterial phyla and function to regulate heme synthesis. Diverse genomes from 978 organisms (924 bacterial and 54 archaeal) were analyzed for the presence of *hemX*. Of these, 113 encode HemX; representative members of this analysis are shown in Fig. 7A. These newly identified homologues expand past the *Bacillales* order, of which representative HemX homologues were previously identified and shown to share function (13). HemX appears to represent an ancient protein family, as it is present in some of the evolutionarily oldest taxa, including *Firmicutes*, *Aquificae*, and *Planctomycetes*. The distribution of *hemX* strongly correlates with the capacity for *de novo* heme synthesis, as *hemX* never occurs in a genome without *gtrR*, and *hemX* never occurs without the capacity for *de novo* heme synthesis. This correlation holds across the microbial kingdom, where *hemX* never occurs in taxa lacking heme biosynthesis genes (within any representatives with sequenced genomes now available): e.g., *Thermotogae*, *Fusobacteria*, and *Mollicutes*. Additionally, the distribution of *hemX* among the members of the *Firmicutes* phylum supports this correlation; *hemX* is present largely in *Bacillales* but does not occur in *Lactobacillales* and only rarely in *Clostridia* (in 2 out 91 genomes analyzed), which is consistent with the frequent capacity for heme synthesis in *Bacillales* relative to *Lactobacillales* and *Clostridia*. Notably, the genomic co-occurrence of *hemX* and *gtrR* holds true in organisms that synthesize heme via any of the 3 heme biosynthetic pathways identified to date: the coproporphyrin-dependent, siroheme-dependent, or classic protoporphyrin-dependent route (Fig. 7A).

Interestingly, HemX is more commonly found in organisms that encode the ability to synthesize both heme and siroheme (see Fig. S8A in the supplemental material) than in organisms that synthesize heme and not siroheme. This suggests that HemX likely impacts siroheme synthesis as a consequence of affecting GtrR abundance by increasing abundance of uroporphyrinogen III, the final shared biosynthetic intermediate. The co-occurrence of *hemX*, g*trR*, and *cysG* is also consistent with our findings in Fig. 6 that siroheme synthesis impacts GtrR abundance.

10.1128/mBio.02287-17.9 FIG S8  *hemX* co-occurs with heme synthesis and shares conserved secondary structure and residues. (A) The genomes shown in Fig. 7 were analyzed for the capacity to synthesize heme or both heme and siroheme, as well as the presence of *hemX*. (B) Alignment of HemX for each of the seven representative organisms, with predicted transmembrane domains in yellow, conserved residues in red, and moderately conserved residues marked with “:” to show conservation among strongly similar amino acids or “.” to show conservation among weakly similar amino acids. Download FIG S8, TIF file, 45.3 MB. Copyright © 2018 Choby et al. 2018 Choby et al. This content is distributed under the terms of the Creative Commons Attribution 4.0 International license.

Next we examined the genomic context of *hemX* homologues across 113 organisms carrying *hemX*, as genes associated with the same pathway or area of metabolism tend to colocalize in prokaryotic genomes (28). In 106 (94%) out of these 113 genomes, the *hemX* and *gtrR* genes are adjacent and likely cotranscribed, which is a very strong indicator of their functional association. Seven representatives of these organisms are shown in Fig. 7B, highlighting the common genomic context of *hemX*, *gtrR*, and other genes involved in uroporphyrinogen synthesis (*hmbS*, *uroS*, *pbgS*, and *gsaM*) and siroheme synthesis (*cysG*).

### HemX homologues share predicted membrane topology and residues.

Comparative genome analysis identified several contextual characteristics of HemX homologues. We therefore investigated the sequences of representative HemX homologs. A multiple-sequence alignment revealed relatively low overall identity among HemX sequences (Fig. 7B); however, the alignment presented in Fig. S8B shows that each HemX homologue shares the same predicted eight-transmembrane-domain topology with N- and C-termini predicted to be extracytoplasmic. Additionally, these divergent homologues share four conserved residues, all in predicted transmembrane domains. Taken together with comparative genome analysis, identification of HemX across bacteria uncovered a strong correlation between *gtrR*, *hemX*, and *de novo* heme synthesis, suggesting that HemX control of GtrR to modulate heme synthesis is a common regulatory strategy among bacteria.

## DISCUSSION

In this report, we identify GtrR abundance as a critical regulator of *S. aureus* heme biosynthesis. GtrR catalyzes the initial step in the heme biosynthetic pathway and is maintained at low levels in WT cells proficient for heme biosynthesis, but specifically increases in response to heme deficiency (Fig. 1B). In this study, we used heme auxotrophs to stimulate production of GtrR (Fig. 1D), but we would predict that in particular niches during infection, an increase in heme synthesis is required and GtrR abundance increases to accommodate this need. Host-imposed nitrosative stress, oxidative stress, and hypoxia, for example, all cause *S. aureus* to increase expression of heme-dependent cytochrome oxidases, catalase, and nitric oxide synthase (29–31).

The drastic difference in GtrR enrichment between the heme auxotroph *pbgS* and Δ*chdC* mutants (Fig. 1D and 5A), with deficits in genes at the beginning and end of the heme biosynthetic pathway, respectively, suggests that GtrR abundance during heme deficiency could be impacted by mechanisms other than heme availability. This is an interesting observation in light of the comparative genome analysis of *hemX*, which suggests that HemX could impact siroheme synthesis as well as heme synthesis. It is possible that GtrR abundance is impacted by differences in heme or siroheme abundance as well as abundance in earlier precursor levels, explaining the difference in GtrR levels in the *pbgS* strain relative to the Δ*chdC* strain.

In addition to the impact of heme deficiency on GtrR, we also identify HemX as a key regulator of GtrR in heme-proficient cells (Fig. 2A). Our broad genomic analysis has identified HemX homologues across bacterial phyla, suggesting that HemX control of heme synthesis via GtrR is a conserved strategy (Fig. 7; Fig. S8). This finding is consistent with the model set forth by Hederstedt and colleagues (18, 21); in both *B. subtilis* and now *S. aureus*, it appears that HemX regulates GtrR abundance posttranscriptionally (Fig. 2B) through an as-yet-undefined mechanism. *B. subtilis* HemX is sufficient to affect GtrR abundance when both are expressed ectopically in *Escherichia coli* (18); however, the contribution of heme or conserved *E. coli* proteins in this system is unclear, making it challenging to conclude if HemX directly interacts with GtrR. Together, our findings are consistent with a model whereby GtrR is regulated by heme abundance and HemX through a multiprotein mechanism. Our finding that GtrR abundance is reduced in the *ΔhemX pbgS* strain relative to the *pbgS* strain and not increased supports this model: heme and HemX both do not directly repress GtrR levels (Fig. 5A). In *Salmonella* strains, which do not encode HemX, GtrR is regulated by N-terminal proteolysis by ClpAP and Lon proteases to keep levels low (32). Additionally, *Salmonella* GtrR binds excess heme through a Cys-170 residue (33). Mutagenesis of the N-terminus degradation sequence or heme binding cysteine disrupts regulation, and it has been proposed that heme-bound GtrR but not apo-GtrR is a substrate for the proteases (32, 34). The mechanism by which *Salmonella* GtrR is regulated by proteases via its N terminus and heme binding is not fully understood, and these regulatory amino acids are not conserved in *S. aureus* GtrR. Likewise, further work is needed to dissect the unique regulatory effects of heme and HemX on GtrR levels and the potential involvement of proteolysis in this process in *S. aureus*.

In the absence of HemX, *S. aureus* synthesizes excess heme. The increase in heme synthesis disrupts intracellular iron homeostasis (Fig. 4), which could additionally disrupt the expression of the staphyloferrin B siderophore biosynthetic genes, which were recently identified to be under the control of a transcriptional activator that is inactive when bound to heme (35). This altered iron homeostasis would likely reduce the function of many important Fe-S cluster-containing enzymes critical to staphylococcal physiology. Additionally, excess heme synthesized in the Δ*hemX* mutant activates the heme stress response (Fig. 3). While activation of the HssRS two-component system was first recognized as the result of exogenous heme, our findings add to a growing body of literature that supports a model whereby endogenous heme and exogenous heme both contribute to HssRS activation and heme toxicity. We have previously identified small molecule activators of heme synthesis that increase intracellular heme and activate HssRS (36–38), adding to our genetic evidence presented in this work that endogenous heme activates HssRS. Here, the increase in endogenous heme in the Δ*hemX* mutant is not toxic because of the HssRS-HrtAB detoxification response. Rather, endogenous heme activation of HssRS provides resistance to heme toxicity through preadaptation and expression of *hrtAB* (Fig. 3C and D). The impact of inactivation of *hemX* on the fitness of pathogens that do not encode the HssRS-HrtAB system would offer insight into whether dysregulated heme synthesis is sufficient to induce heme toxicity from within.

This study found that regulation of GtrR abundance is sufficient to regulate total heme synthesis (Fig. 2), consistent with multiple reports that ALA formation is a critical rate-limiting step in heme synthesis (14). Indeed, regulation of ALA synthesis via control of either GtrR or ALAS has emerged as a theme across kingdoms. In metazoans, two ALAS isoforms exist and are impacted by heme (reviewed in reference 39). In the plant model organism *Arabidopsis thaliana*, ALA synthesis is regulated by degradation of GluTR via Clp proteolysis from the N terminus as well as stabilization and activation of a regulatory binding protein (40, 41). In Gram-negative model organisms, as mentioned above, GtrR abundance is regulated by heme and proteolysis (32). Our findings extend this paradigm further into the *Firmicutes* phylum of bacteria.

The specific mechanism by which HemX impacts GtrR abundance remains undefined. HemX is annotated as a member of the cytochrome *c* assembly protein family (Pfam accession no. PF01578), suggesting that it may be involved in heme binding and trafficking at the membrane. However, *S. aureus* does not encode *c*-type cytochromes. The capacity of HemX to bind heme has not been experimentally validated, but excess heme does accumulate in the membrane (42), which is suggestive of a potential role for membrane-localized heme reservoirs or chaperones. The limited regions of HemX predicted to be cytoplasmic suggest that protein-protein interactions likely occur between other membrane proteins, but no HemX-interacting partners have been identified to date. Additionally, GtrR residues that impact regulation by either heme or HemX are unknown, but would offer information as to the regulatory steps between heme, HemX, and GtrR, which appear to be complex. Although heme-dependent inhibition of *S. aureus* heme synthesis was first proposed in 1962 (43), the impact of HemX and heme on GtrR abundance continues to warrant further investigation.

## MATERIALS AND METHODS

### Bacterial strains and reagents.

Bacterial strains (Table 1), plasmids (see Table S1 in the supplemental material), and primers (Table S1) are listed in the specified table. *S. aureus* strains were grown routinely on tryptic soy agar (TSA) or broth (TSB) supplemented with 10 µg/ml chloramphenicol or 10 µg/ml erythromycin when necessary. When used, heme (hemin chloride) was used at the concentrations noted. Heme was prepared fresh at 10 mM in 0.1 M NaOH; for experiments in which heme was used, an equal volume of 0.1 M NaOH was used for all conditions. *E. coli* strains were grown on lysogeny broth (LB) or LB agar (LBA), supplemented with 50 µg/ml carbenicillin when necessary. For growth in liquid medium, an Innova44 incubator shaking at 180 rpm was used. For standard cultures of 4 to 5 ml, 15-ml round-bottomed polypropylene tubes with aeration lids were used, at a 45° angle in the incubator. For cloning and mutagenesis in plasmids, all constructs were confirmed by sequencing (GeneWiz). Unless noted otherwise, all chemicals are from Sigma. All molecular biology reagents were from New England Biolabs (NEB) and used according to the manufacturer’s instructions, unless otherwise noted. Phusion 2X Hi-fidelity master mix was used for all PCRs for cloning. As necessary, plasmids were transformed by electroporation from *E. coli* into the *S. aureus* cloning intermediate strain RN4220 before isolation and subsequent electroporation into the final *S. aureus* strains.

10.1128/mBio.02287-17.10 TABLE S1  Plasmids and primers used in this study. Download TABLE S1, DOCX file, 0.1 MB. Copyright © 2018 Choby et al. 2018 Choby et al. This content is distributed under the terms of the Creative Commons Attribution 4.0 International license.

**TABLE 1:** *S. aureus* strains used in this study

Strain	Genotype	Description	Source or reference
Newman	WT	Wild-type, methicillin-sensitive clinical isolate	57
Newman	*pbgS*	*pbgS* (*NWMN_1562*) gene interrupted with erythromycin resistance gene *ermB* by homologous recombination, transduced into Newman	3
Newman	Δ*chdC*	In-frame unmarked deletion of *chdC* (*NWMN_0550*) generated by allelic exchange	58
Newman	Δ*hemX* Δ*chdC*	In-frame unmarked deletion of *chdC* (*NWMN_0550*) generated by allelic exchange in Δ*hemX* strain	This work
Newman	Δ*menB*	In-frame unmarked deletion of *menB* generated by allelic exchange	59
Newman	Δ*qoxB cydB*	In-frame unmarked deletion of *qoxB* and *cydB*::Tn	3
JE2	*katA*	*katA*::Tn (*NE1366*)	BEI (46)
Newman	*katA*	*katA*::Tn (*NE1366*), transduced into Newman	This work
Newman	Δ*hemX*	In-frame unmarked deletion of *hemX* (*NWMN_1565*)	This work
RN9011		RN4220 carrying pRN7023 integrase plasmid	24
RN9011	*attC*::P_*lgt*_	pJC1111 P_*lgt*_ integrated into chromosome at *attC* locus	This work
RN9011	*attC*::P*_lgt_hemX*	pJC1111 P_lgt_*hemX* integrated into chromosome at *attC* locus	This work
Newman	Δ*hemX attC*::P_*lgt*_	pJC1111 P_*lgt*_ integrated into chromosome at *attC* locus	This work
Newman	Δ*hemX attC*::P*_lgt_hemX*	pJC1111 P*_lgt_hemX* integrated into chromosome at *attC* locus	This work
Newman	*attC*::P*_lgt_*	pJC1111 P_*lgt*_ integrated into chromosome at *attC* locus	This work
Newman	Δ*hemX pbgS*	In-frame unmarked deletion of *hemX* (*NWMN_1565*) in *pbgS* strain	This work
Newman	Δ*hssRS*	In-frame unmarked deletion of *hssRS* generated by allelic exchange	60
Newman	Δ*hrtB*	In-frame unmarked deletion of *hrtB* generated by allelic exchange	61
Newman	*hrtB*	*hrtB*::Tn(PhiNE01762)	10
Newman	Δ*hemX hrtB*	*hrtB*::Tn(PhiNE01762) allele transduced to Δ*hemX* strain	This work
JE2	*cysG*	*cysG*::Tn (*NE1931*; *SAUSA300_2553*::Tn)	BEI (46)
Newman	*cysG*	*cysG*::Tn (*NE1931*) transduced into Newman	This work
Newman	Δ*hemX cysG*	*cysG*::Tn (*NE1931*) transduced into Newman Δ*hemX*	This work
JE2	*nirD*	*nirD*::Tn (*NE1279*)	BEI (46)
Newman	*nirD*	*nirD*::Tn (*NE1279*) transduced into Newman	This work
Newman	Δ*hemX nirD*	*nirD*::Tn (*NE1279*) transduced into Newman Δ*hemX*	This work
Newman	Δ*gtrR-hemX*	In-frame unmarked deletion of *gtrR* and *hemX* (*NWMN_1565-1566*) generated by single allelic exchange	This work
RN4220		Restriction-deficient cloning intermediate strain	62

### (i) Deletion of genes by allelic exchange.

Deletion of *hemX* and *chdC* was performed by allelic exchange as described in reference 44 with some modifications. The pKOR1 plasmids containing ~1-kb homologous regions flanking upstream and downstream of the gene to be deleted were prepared using NEB Hi-Fi assembly according to manufacturer’s suggestions. The pKOR1 backbone was amplified by PCR using JC291/292, which produces a linear product not including the *attB* recombination sites. The ~1-kb flanking regions were amplified from the *S. aureus* Newman genomic DNA. Deletions were confirmed by PCR using isolated genomic DNA and complemented by providing the gene in *cis* or *trans*. Additional details are found in Text S1 in the supplemental material.

10.1128/mBio.02287-17.1 TEXT S1  Supplemental materials and methods and references. Download TEXT S1, DOCX file, 0.1 MB. Copyright © 2018 Choby et al. 2018 Choby et al. This content is distributed under the terms of the Creative Commons Attribution 4.0 International license.

### (ii) *hemX* chromosomal integration.

Chromosomal complementation was performed by cloning P_*lgt*_ or P*_lgt_hemX* into pJC1111. P_*lgt*_ was PCR amplified from pOS1 P_*lgt*_ using JC158/229 and subsequently cloned into the multiple cloning site of pJC1111 after restriction digestion with SalI and BamHI. *hemX* was cloned into pOS1 P_*lgt*_ by amplifying *hemX* flanked by NdeI and BamHI sites from *S. aureus* Newman genomic DNA using primers JC157/155 and ligated (T4 ligase) into the multiple cloning site of pOS1 P_*lgt*_ after restriction digestion with NdeI and BamHI. P*_lgt_hemX* was amplified from pOS1 P*_lgt_hemX* using JC158/155 and subsequently cloned into the SalI and BamHI sites of pJC1111 after restriction digestion with SalI and BamHI. pJC1111 P_*lgt*_ and pJC1111 P*_lgt_hemX* were integrated into the chromosome of strain RN9011 as described previously (24) and then transduced into the *S. aureus* Newman WT or Δ*hemX* mutant as noted. Transductions of pJC111 loci were performed with φ85 as described in reference 45, with some modifications: after incubation of donor phage with recipient strains and washing with sodium citrate, cells were allowed to recover for 4 h in TSB with 40 mM sodium citrate at 37°C with shaking and plated to TSA containing 0.15 mM cadmium chloride.

### (iii) Transduction of transposon library alleles.

For transduction of transposon library alleles, the *katA*::Tn (*NE1366*), *cysG*::Tn (*NE1931*), *nirD*::Tn (*NE1279*), and *hrtB*::Tn (PhiNE01762) transposon alleles were transduced to the *S. aureus* Newman and Δ*hemX* strains as listed in Table 1 as described previously (45) using bacteriophage φ85; alleles were confirmed by an inverse-PCR method and Sanger sequencing (46).

### Catalase activity.

To assess catalase activity, strains were grown for 16 h in TSB, and then 50 µl of each culture was spotted onto a TSA plate and streaked for isolation. After 24 h of growth at 37°C, 50 µl of 30% H_2_O_2_ was added to each strain and immediately imaged.

### LC-MRM-MS/MS.

Strains were streaked onto TSA and grown for 24 h at 37°C. Cultures were started from single colonies in 5 ml of RPMI plus 1% Casamino Acids and grown at 37°C for 15 h. Overnight cultures were subcultured 1:100 into RPMI plus 1% Casamino Acids and grown until mid-exponential phase. For small-colony variants without chemical complementation, overnight cultures were subcultured 1:25. For conditions under which heme was added, 2 µM was used; for menaquinone, 12.5 µM menaquinone-vitamin K_2_ was used.

For anaerobic experiments, a Coy (Grass Lake, MI) anaerobic chamber was used, filled with a mixture of 90% nitrogen, 5% carbon dioxide, and 5% hydrogen gases, and hydrogen levels were monitored to ensure a minimum 2% hydrogen concentration. Palladium catalysts (Coy) were used to remove any residual oxygen by reaction with hydrogen. A Coy static incubator was maintained at 37°C. Solutions and plasticware were allowed to equilibrate for >24 h inside the glove box before use. For anaerobic samples, strains were streaked onto TSA and grown aerobically for 24 h at 37°C. Cultures were started from single colonies in 5 ml of anaerobic TSB and grown at 37°C for 15 h. Overnight cultures were subcultured 1:100 into anaerobic TSB containing 40 mM sodium nitrate and grown until the mid-exponential phase. Protein was collected, tryptically digested, and subjected to LC-MRM-MS/MS as described in Text S1 in the supplemental material.

### Anaerobic growth curves.

The *S. aureus* Newman WT, Δ*hemX*, *cysG*, Δ*hemX cysG*, *nirD*, and Δ*hemX nirD* strains were streaked onto TSA and grown aerobically for 24 h at 37°C. Cultures were started from single colonies in 3 ml of anaerobic TSB and grown at 37°C for 15 h. Overnight cultures were subcultured 1:200 in round-bottomed 96-well plates with 200 μl of anaerobic TSB containing 40 mM sodium nitrate or an equal volume of sterile water and covered with Breathe-Easy gas-permeable seal (Sigma). Growth was monitored by optical density (OD) over time in a BioTek Synergy H1.

### Quantitative reverse transcriptase PCR.

Strains were streaked onto TSA and grown at 24 h at 37°C. Cultures were started from single colonies in 5 ml of RPMI plus 1% Casamino Acids and grown at 37°C for 15 h. Overnight cultures were subcultured 1:100 (WT and Δ*hemX* mutant) or 1:25 (*pbgS* Δ*hemX pgbS* strain) into RPMI plus 1% Casamino acids and grown until the mid-exponential phase. An equal volume of ice-cold acetone-ethanol was added, and the mixture was stored at −80°C. RNA was isolated using Tri reagent and chloroform and precipitated with isopropanol. Isolated RNAs were treated with DNase I (Thermo) according to the manufacturer’s instructions, and RNA was reisolated using Qiagen RNeasy kit. cDNA was synthesized from 2 µg of RNA by incubation with Moloney murine leukemia virus (MMLV) reverse transcriptase (Thermo), using transcript-specific primers (JC83/84 for *gyrA*, JC53/54 for *gtrR*, and JC55/56 for *gsaM*). Quantitative PCR (qPCR) was performed using SYBR green (Thermo) according to the manufacturer’s instructions, using primers JC81/82 for *gyrA*, HS1/2 for *gtrR*, and HS for *gsaM*. Transcript abundance was quantified using the threshold cycle (ΔΔ*C*_*T*_) method after normalization to *gyrA* abundance.

### ALA quantification.

ALA quantification was modified from reference 47. The *S. aureus* WT and Δ*hemX* mutant strains were streaked onto TSA and grown for 18 h at 37°C. Single colonies were used to start 5-ml cultures in TSB and grown for 12 h at 37°C, then 1 ml was inoculated into 100 ml of TSB in a 250-ml Erlenmeyer flask and grown at 37°C for 14 h. The cell wall was removed by incubation in TSM (100 mM Tris-Cl, pH 7, 500 mM sucrose, 10 mM MgCl_2_) plus 40 µg/ml lysostaphin and incubated at 37°C for 45 min. Protoplasts were collected by centrifugation and resuspended in 1 ml 10% trichloroacetic acid (TCA). Samples were incubated on ice and intermittently lysed by sonication. The soluble fraction was collected by centrifugation and neutralized to pH 7 with 6 M NaOH, then added to a Dowex 1x-4 resin in column converted to the acetate form before use. In this form, the column retains porphobilinogen but allows ALA to flow through. Six hundred microliters of flowthrough was added to 200 µl of 8% acetyl acetone in 2 M sodium acetate buffer, incubated for 15 min at 90°C to form the pyrrolic condensation product, and cooled to room temperature. Five hundred microliters of sample was added to 500 µl of modified Ehrlich’s reagent and incubated for 10 min at room temperature, and the resulting absorbance was measured at 552 and 650 nm in a Cary 50 Bio UV-visible (UV-Vis) spectrophotometer. The relative concentration of ALA was calculated based on an extinction coefficient of 7.2 × 10^−4^ M^−1^ cm^−1^.

### LC-qTOF-MS porphyrin quantification.

Porphyrins were extracted from the *S. aureus* WT and Δ*hemX* strains grown to the stationary phase and analyzed by LC-qTOF-MS described in detail in Text S1 in the supplemental material.

### Pyridine hemochromagen quantification.

Strains were streaked onto TSA and grown for 18 h at 37°C. Single colonies were used to start 5-ml cultures of TSB and grown at 37°C for 10 h. Sixty microliters of each culture was added to 6 ml of TSB and grown for 16 h at 37°C. Cells were collected by centrifugation, and the cell wall was removed by incubation in 20 mM potassium phosphate buffer (pH 7.4) containing 20 µg of lysostaphin for 45 min at 37°C. Samples were lysed by sonication, and unbroken cells were collected by centrifugation. Four hundred fifty microliters of the soluble supernatant was added to 450 µl of 0.2 M NaOH containing 40% pyridine and 500 µM potassium ferricyanide. Absorbance was measured in a Cary 50 Bio UV-Vis spectrophotometer from 540 to 590 nm. Ten microliters of 0.5 M sodium dithionite prepared in 0.5 M NaOH was added to samples, the mixture was incubated for 5 min, and absorbance (*A*) was measured again from 540 to 590 nm. Heme quantity is calculated using Δ*A* = (*A*_557 reduced_ − *A*_557 oxidized_) − (*A*_575 reduced_ − *A*_575__oxidized_) and an extinction coefficient of 32.4 mM^−1^ cm^−1^.

### Bioluminescent reporter assay.

The *S. aureus* WT and Δ*hemX* strain with pXen-1 or P*_hrt_luxABCDE* were streaked onto TSA-chloramphenicol prepared with 0 or 20 µM heme. After 18 h, the plates were imaged using a Xenogen IVIS 2000.

### XylE reporter assay.

XylE abundance in cellular lysate was assessed spectrophotometrically by measuring formation of 2-hydroxymuconic acid from catechol after growth in TSB containing chloramphenicol and 0 to 2 µM heme, as described previously (10).

### Heme killing assay.

*S. aureus* WT, Δ*hemX*, and Δ*hrtB* strains were streaked onto TSA and grown for 24 h at 37°C. Single colonies were used to start 5-ml cultures of TSB and grown at 37°C for 14 h. Two microliters of each culture was added to 148 µl of TSB containing different concentrations of heme in a 96-well round-bottomed plate and incubated at 37°C for 2 h. Samples were serially diluted in phosphate-buffered saline (PBS) and plated to TSA for CFU enumeration after 24 h of growth at 37°C.

### Heme toxicity growth curves.

Strains were streaked onto TSA and grown for 24 h at 37°C. Single colonies were used to start 5 ml cultures of TSB and grown for 16 h at 37°C containing 0 or 2 µM heme as noted. One microliter of each culture was added to 199 µl of medium containing 0 or 10 µM heme, as noted, in a 96-well round-bottomed plate, and growth was monitored over time at 37°C by measuring the optical density at 600 nm (OD_600_) in a BioTek Synergy2 spectrophotometer and analyzed with BioTek Gen5 software.

### Growth in minimal medium.

Chemically defined media (CDM) supplemented with 5 mg/ml glucose was prepared as previously described (48), with the exception that iron was not added. Strains were streaked onto TSA and grown for 24 h at 37°C. Single colonies were used to start 5-ml cultures in TSB and grown for 14 h at 37°C. Cells were collected by centrifugation, washed in PBS twice, and then resuspended in 5 ml of PBS. One microliter was added to 199 µl of CDM containing 1 µM ethylenediamine-*N*,*N*′-bis(2-hydroxyphenylacetic acid) (EDDHA; LGC Standards) or an equal volume of 0.1 M NaOH (vehicle) in a 96-well round-bottomed plate. Growth was monitored for 24 h with shaking at 37°C in a BioTek EPOCH2 spectrophotometer and analyzed with BioTek Gen5 software.

### pOS1 P*_isdA_gfp* reporter assay.

The *S. aureus* WT pOS1 P*_isdA_gfp* and Δ*hemX* pOS1 P*_isdA_gfp* strains were streaked onto TSA-chloramphenicol and grown for 14 h at 37°C. Single colonies were used to inoculate 5-ml cultures of TSB-chloramphenicol and grown at 37°C for 8.5 h. One microliter of each culture was used to inoculate 199 µl of TSB-chloramphenicol containing 1 mM 2,2-dipyridyl or an equal volume of ethanol (vehicle). Growth was monitored over the course of 16 h by measuring OD_600_ as well as relative fluorescence at 485 nm (excitation) and 510 nm (emission) in a BioTek Cytation5 spectrophotometer and analyzed with BioTek Gen5 software.

### Comparative genome analysis.

With over 100,000 prokaryotic genomes currently available in public databases and many more in the pipelines (http://www.genomesonline.org), it is not practical or possible to perform meaningful comparative analysis on all of them simultaneously. Thus, a set of diverse representative prokaryotic genomes have been developed in the SEED database as follows. The algorithm for computing molecular operational taxonomic units (OTU) based on DNA barcode data (49, 50) was used to group ~12,600 prokaryotic genomes available in the SEED database in October 2013 into about 1,000 taxon groups. One or two representative genomes (rarely three) for each OTU were selected based on the largest amount of published experimental data and the highest level of research interest within the scientific community. The resultant collection of 982 diverse genomes (928 eubacterial and 54 archaeal) creates a manageable set that accurately represents the immense diversity of the over 12,000 prokaryotic organisms with sequenced genomes. Importantly, it is not skewed by an overabundance of genomes for a few microbial genera (medically or industrially important), such as *Enterobacteriaceae*, streptococci, mycobacteria, etc.

The HemX protein family was exhaustively annotated for this set of 982 representative microbial genomes in the SEED database (51). Contextual associations for this family were predicted based on the patterns of co-occurrence and/or colocalization of its members with other protein families using the set of tools for comparative genome analysis available in SEED (52) within the functional and genomic contexts provided by the subsystem “Heme Biosynthesis: protoporphyrin-, coproporphyrin- and siroheme-dependent pathways” (http://pubseed.theseed.org//SubsysEditor.cgi?page=ShowSubsystem&subsystem=Heme_Biosynthesis%3A_protoporphyrin-%2C_coproporphyrin-_and_siroheme-dependent_pathways). Phylogenetic distribution of the HemX protein family was mapped onto the tree of life (53), and protoheme biosynthetic pathway analysis was adapted from reference 14.

### HemX multiple sequence alignment and topology prediction.

The HemX multiple sequence alignment was KEGG ClustalW (http://www.genome.jp [accessed March 2017]) (54) using *Staphylococcus aureus* strain Newman, *Staphylococcus epidermidis* strain ATCC 12228, *Bacillus anthracis* strain Sterne, *Chlorobium tepidum* strain TLS, *Aquifex aeolicus* strain VF5, *Desulfovfibrio vulgaris* strain DP4, and *Geobacter sulfurreducens* strain PCA. The transmembrane domains depicted were predicted by MEMSAT3 (http://bioinf.cs.ucl.ac.uk; accessed March 2017) as described previously (55). All models were confirmed using TMHMM 2.0 (http://www.cbs.dtu.dk [accessed March 2017]) (56), and predictions were matched across prediction servers, with the exception of *Chlorobium tepidum*, which TMHMM2.0 predicts to have seven rather than eight transmembrane domains.

### Statistical analysis.

All data analysis and statistical tests were performed using GraphPad Prism 6 software. Replicate numbers and statistical tests for each experiment are listed in the figure legends.

## References

[1] Choby JE, Skaar EP 2016 Heme synthesis and acquisition in bacterial pathogens. J Mol Biol 428:3408–3428. doi:10.1016/j.jmb.2016.03.018. 27019298 PMC5125930

[2] Klevens RM, Morrison MA, Nadle J, Petit S, Gershman K, Ray S, Harrison LH, Lynfield R, Dumyati G, Townes JM, Craig AS, Zell ER, Fosheim GE, McDougal LK, Carey RB, Fridkin SK, Active Bacterial Core surveillance (ABCs) MRSA Investigators 2007 Invasive methicillin-resistant Staphylococcus aureus infections in the United States. JAMA 298:1763–1771. doi:10.1001/jama.298.15.1763. 17940231

[3] Hammer ND, Reniere ML, Cassat JE, Zhang Y, Hirsch AO, Hood MI, Skaar EP 2013 Two heme-dependent terminal oxidases power *Staphylococcus aureus* organ-specific colonization of the vertebrate host. mBio 4:e00241-13. doi:10.1128/mBio.00241-13. 23900169 PMC3735196

[4] Hammer ND, Schurig-Briccio LA, Gerdes SY, Gennis RB, Skaar EP 2016 CtaM is required for menaquinol oxidase aa3 function in *Staphylococcus aureus*. mBio 7:e00823-16. doi:10.1128/mBio.00823-16. 27406563 PMC4958251

[5] Dailey HA, Gerdes S, Dailey TA, Burch JS, Phillips JD 2015 Noncanonical coproporphyrin-dependent bacterial heme biosynthesis pathway that does not use protoporphyrin. Proc Natl Acad Sci U S A 112:2210–2215. doi:10.1073/pnas.1416285112. 25646457 PMC4343137

[6] Lobo SA, Scott A, Videira MA, Winpenny D, Gardner M, Palmer MJ, Schroeder S, Lawrence AD, Parkinson T, Warren MJ, Saraiva LM 2015 *Staphylococcus aureus* haem biosynthesis: characterisation of the enzymes involved in final steps of the pathway. Mol Microbiol 97:472–487. doi:10.1111/mmi.13041. 25908396

[7] Cosgrove K, Coutts G, Jonsson IM, Tarkowski A, Kokai-Kun JF, Mond JJ, Foster SJ 2007 Catalase (KatA) and alkyl hydroperoxide reductase (AhpC) have compensatory roles in peroxide stress resistance and are required for survival, persistence, and nasal colonization in *Staphylococcus aureus*. J Bacteriol 189:1025–1035. doi:10.1128/JB.01524-06. 17114262 PMC1797328

[8] van Sorge NM, Beasley FC, Gusarov I, Gonzalez DJ, von Köckritz-Blickwede M, Anik S, Borkowski AW, Dorrestein PC, Nudler E, Nizet V 2013 Methicillin-resistant *Staphylococcus aureus* bacterial nitric-oxide synthase affects antibiotic sensitivity and skin abscess development. J Biol Chem 288:6417–6426. doi:10.1074/jbc.M112.448738. 23322784 PMC3585076

[9] Mogen AB, Carroll RK, James KL, Lima G, Silva D, Culver JA, Petucci C, Shaw LN, Rice KC 2017 *Staphylococcus aureus* nitric oxide synthase (saNOS) modulates aerobic respiratory metabolism and cell physiology. Mol Microbiol 105:139–157. doi:10.1111/mmi.13693. 28431199 PMC5641370

[10] Torres VJ, Stauff DL, Pishchany G, Bezbradica JS, Gordy LE, Iturregui J, Anderson KL, Dunman PM, Joyce S, Skaar EP 2007 A *Staphylococcus aureus* regulatory system that responds to host heme and modulates virulence. Cell Host Microbe 1:109–119. doi:10.1016/j.chom.2007.03.001. 18005689 PMC2083280

[11] Beale SI, Castelfranco PA 1973 ^14^C incorporation from exogenous compounds into δ-aminolevulinic acid by greening cucumber cotyledons. Biochem Biophys Res Commun 52:143–149. doi:10.1016/0006-291X(73)90966-2. 4712185

[12] Schön A, Krupp G, Gough S, Berry-Lowe S, Kannangara CG, Söll D 1986 The RNA required in the first step of chlorophyll biosynthesis is a chloroplast glutamate tRNA. Nature 322:281–284. doi:10.1038/322281a0. 3637637

[13] Moser J, Schubert WD, Beier V, Bringemeier I, Jahn D, Heinz DW 2001 V-shaped structure of glutamyl-tRNA reductase, the first enzyme of tRNA-dependent tetrapyrrole biosynthesis. EMBO J 20:6583–6590. doi:10.1093/emboj/20.23.6583. 11726494 PMC125327

[14] Dailey HA, Dailey TA, Gerdes S, Jahn D, Jahn M, O’Brian MR, Warren MJ 2017 Prokaryotic heme biosynthesis: multiple pathways to a common essential product. Microbiol Mol Biol Rev 81:81:e00048-16. doi:10.1128/MMBR.00048-16. PMC5312243 28123057

[15] Hansson M, Gustafsson MC, Kannangara CG, Hederstedt L 1997 Isolated *Bacillus subtilis* HemY has coproporphyrinogen III to coproporphyrin III oxidase activity. Biochim Biophys Acta 1340:97–104. doi:10.1016/S0167-4838(97)00030-7. 9217019

[16] Gerber SA, Rush J, Stemman O, Kirschner MW, Gygi SP 2003 Absolute quantification of proteins and phosphoproteins from cell lysates by tandem MS. Proc Natl Acad Sci U S A 100:6940–6945. doi:10.1073/pnas.0832254100. 12771378 PMC165809

[17] Proctor RA, von Eiff C, Kahl BC, Becker K, McNamara P, Herrmann M, Peters G 2006 Small colony variants: a pathogenic form of bacteria that facilitates persistent and recurrent infections. Nat Rev Microbiol 4:295–305. doi:10.1038/nrmicro1384. 16541137

[18] Schröder I, Johansson P, Rutberg L, Hederstedt L 1994 The *hemX* gene of the *Bacillus subtilis hemAXCDBL* operon encodes a membrane protein, negatively affecting the steady-state cellular concentration of HemA (glutamyl-tRNA reductase). Microbiology 140:731–740. doi:10.1099/00221287-140-4-731. 8012594

[19] Wang LY, Brown L, Elliott M, Elliott T 1997 Regulation of heme biosynthesis in *Salmonella typhimurium*: activity of glutamyl-tRNA reductase (HemA) is greatly elevated during heme limitation by a mechanism which increases abundance of the protein. J Bacteriol 179:2907–2914. doi:10.1128/jb.179.9.2907-2914.1997. 9139907 PMC179053

[20] Bibb LA, Kunkle CA, Schmitt MP 2007 The ChrA-ChrS and HrrA-HrrS signal transduction systems are required for activation of the *hmuO* promoter and repression of the *hemA* promoter in *Corynebacterium diphtheriae*. Infect Immun 75:2421–2431. doi:10.1128/IAI.01821-06. 17353293 PMC1865786

[21] Johansson P, Hederstedt L 1999 Organization of genes for tetrapyrrole biosynthesis in Gram-positive bacteria. Microbiology 145:529–538. doi:10.1099/13500872-145-3-529. 10217486

[22] Hansson M, Rutberg L, Schröder I, Hederstedt L 1991 The *Bacillus subtilis hemAXCDBL* gene cluster, which encodes enzymes of the biosynthetic pathway from glutamate to uroporphyrinogen III. J Bacteriol 173:2590–2599. doi:10.1128/jb.173.8.2590-2599.1991. 1672867 PMC207825

[23] Schröder I, Hederstedt L, Kannangara CG, Gough P 1992 Glutamyl-tRNA reductase activity in *Bacillus subtilis* is dependent on the *hemA* gene product. Biochem J 281:843–850. doi:10.1042/bj2810843. 1536660 PMC1130766

[24] Chen J, Yoong P, Ram G, Torres VJ, Novick RP 2014 Single-copy vectors for integration at the SaPI1 attachment site for *Staphylococcus aureus*. Plasmid 76:1–7. doi:10.1016/j.plasmid.2014.08.001. 25192956 PMC4346540

[25] Hoober JK, Kahn A, Ash DE, Gough S, Kannangara CG 1988 Biosynthesis of delta-aminolevulinate in greening barley leaves. IX. Structure of the substrate, mode of gabaculine inhibition, and the catalytic mechanism of glutamate 1-semialdehyde aminotransferase. Carlsberg Res Commun 53:11–25. doi:10.1007/BF02908411. 3256306

[26] Mazmanian SK, Skaar EP, Gaspar AH, Humayun M, Gornicki P, Jelenska J, Joachmiak A, Missiakas DM, Schneewind O 2003 Passage of heme-iron across the envelope of *Staphylococcus aureus*. Science 299:906–909. doi:10.1126/science.1081147. 12574635

[27] Schlag S, Fuchs S, Nerz C, Gaupp R, Engelmann S, Liebeke M, Lalk M, Hecker M, Götz F 2008 Characterization of the oxygen-responsive NreABC regulon of Staphylococcus aureus. J Bacteriol 190:7847–7858. doi:10.1128/JB.00905-08. 18820014 PMC2583599

[28] Overbeek R, Fonstein M, D’Souza M, Pusch GD, Maltsev N 1999 The use of gene clusters to infer functional coupling. Proc Natl Acad Sci U S A 96:2896–2901. doi:10.1073/pnas.96.6.2896. 10077608 PMC15866

[29] Chang W, Small DA, Toghrol F, Bentley WE 2006 Global transcriptome analysis of *Staphylococcus aureus* response to hydrogen peroxide. J Bacteriol 188:1648–1659. doi:10.1128/JB.188.4.1648-1659.2006. 16452450 PMC1367260

[30] Horsburgh MJ, Clements MO, Crossley H, Ingham E, Foster SJ 2001 PerR controls oxidative stress resistance and iron storage proteins and is required for virulence in *Staphylococcus aureus*. Infect Immun 69:3744–3754. doi:10.1128/IAI.69.6.3744-3754.2001. 11349039 PMC98383

[31] Kinkel TL, Roux CM, Dunman PM, Fang FC 2013 The *Staphylococcus aureus* SrrAB two-component system promotes resistance to nitrosative stress and hypoxia. mBio 4:e00696-13. doi:10.1128/mBio.00696-13. 24222487 PMC3892780

[32] Wang L, Elliott M, Elliott T 1999 Conditional stability of the HemA protein (glutamyl-tRNA reductase) regulates heme biosynthesis in *Salmonella typhimurium*. J Bacteriol 181:1211–1219. 9973348 10.1128/jb.181.4.1211-1219.1999 PMC93499

[33] Jones AM, Elliott T 2010 A purified mutant HemA protein from *Salmonella enterica* serovar Typhimurium lacks bound heme and is defective for heme-mediated regulation *in vivo*. FEMS Microbiol Lett 307:41–47. doi:10.1111/j.1574-6968.2010.01967.x. 20412302

[34] Wang L, Wilson S, Elliott T 1999 A mutant HemA protein with positive charge close to the N terminus is stabilized against heme-regulated proteolysis in *Salmonella typhimurium*. J Bacteriol 181:6033–6041. 10498716 10.1128/jb.181.19.6033-6041.1999 PMC103631

[35] Laakso HA, Marolda CL, Pinter TB, Stillman MJ, Heinrichs DE 2016 A heme-responsive regulator controls synthesis of staphyloferrin B in *Staphylococcus aureus*. J Biol Chem 291:29–40. doi:10.1074/jbc.M115.696625. 26534960 PMC4697164

[36] Dutter BF, Mike LA, Reid PR, Chong KM, Ramos-Hunter SJ, Skaar EP, Sulikowski GA 2016 Decoupling activation of heme biosynthesis from anaerobic toxicity in a molecule active in Staphylococcus aureus. ACS Chem Biol 11:1354–1361. 26890615 10.1021/acschembio.5b00934 PMC5117894

[37] Mike LA, Dutter BF, Stauff DL, Moore JL, Vitko NP, Aranmolate O, Kehl-Fie TE, Sullivan S, Reid PR, DuBois JL, Richardson AR, Caprioli RM, Sulikowski GA, Skaar EP 2013 Activation of heme biosynthesis by a small molecule that is toxic to fermenting *Staphylococcus aureus*. Proc Natl Acad Sci U S A 110:8206–8211. doi:10.1073/pnas.1303674110. 23630262 PMC3657828

[38] Surdel MC, Horvath DJ Jr, Lojek LJ, Fullen AR, Simpson J, Dutter BF, Salleng KJ, Ford JB, Jenkins JL, Nagarajan R, Teixeira PL, Albertolle M, Georgiev IS, Jansen ED, Sulikowski GA, Lacy DB, Dailey HA, Skaar EP 2017 Antibacterial photosensitization through activation of coproporphyrinogen oxidase. Proc Natl Acad Sci U S A 114:E6652–E6659. doi:10.1073/pnas.1700469114 pii: 201700469. PMC5559000 28739897

[39] Girvan HM, Munro AW 2013 Heme sensor proteins. J Biol Chem 288:13194–13203. doi:10.1074/jbc.R112.422642. 23539616 PMC3650359

[40] Zhao A, Fang Y, Chen X, Zhao S, Dong W, Lin Y, Gong W, Liu L 2014 Crystal structure of *Arabidopsis* glutamyl-tRNA reductase in complex with its stimulator protein. Proc Natl Acad Sci U S A 111:6630–6635. doi:10.1073/pnas.1400166111. 24753615 PMC4020052

[41] Apitz J, Nishimura K, Schmied J, Wolf A, Hedtke B, van Wijk KJ, Grimm B 2016 Posttranslational control of ALA synthesis includes GluTR degradation by Clp protease and stabilization by GluTR-binding protein. Plant Physiol 170:2040–2051. doi:10.1104/pp.15.01945. 26884485 PMC4825132

[42] Skaar EP, Humayun M, Bae T, DeBord KL, Schneewind O 2004 Iron-source preference of *Staphylococcus aureus* infections. Science 305:1626–1628. doi:10.1126/science.1099930. 15361626

[43] Jensen J 1962 The effect of heme on tetrapyrrol synthesis in a heme requiring *Staphylococcus aureus*. Biochem Biophys Res Commun 8:271–277. doi:10.1016/0006-291X(62)90276-0. 14451646

[44] Bae T, Schneewind O 2006 Allelic replacement in *Staphylococcus aureus* with inducible counter-selection. Plasmid 55:58–63. doi:10.1016/j.plasmid.2005.05.005. 16051359

[45] Choby JE, Mike LA, Mashruwala AA, Dutter BF, Dunman PM, Sulikowski GA, Boyd JM, Skaar EP 2016 A small-molecule inhibitor of iron-sulfur cluster assembly uncovers a link between virulence regulation and metabolism in *Staphylococcus aureus*. Cell Chem Biol 23:1351–1361. doi:10.1016/j.chembiol.2016.09.012. 27773628 PMC5117899

[46] Fey PD, Endres JL, Yajjala VK, Widhelm TJ, Boissy RJ, Bose JL, Bayles KW 2013 A genetic resource for rapid and comprehensive phenotype screening of nonessential *Staphylococcus aureus* genes. mBio 4:e00537-12. doi:10.1128/mBio.00537-12. 23404398 PMC3573662

[47] Kardon JR, Yien YY, Huston NC, Branco DS, Hildick-Smith GJ, Rhee KY, Paw BH, Baker TA 2015 Mitochondrial ClpX activates a key enzyme for heme biosynthesis and erythropoiesis. Cell 161:858–867. doi:10.1016/j.cell.2015.04.017. 25957689 PMC4467794

[48] Vitko NP, Richardson AR 2013 Laboratory maintenance of methicillin-resistant *Staphylococcus aureus* (MRSA). Curr Protoc Microbiol Chapter 9:Unit 9C.2. doi:10.1002/9780471729259.mc09c02s28. PMC4070006 23408135

[49] Blaxter M, Mann J, Chapman T, Thomas F, Whitton C, Floyd R, Abebe E 2005 Defining operational taxonomic units using DNA barcode data. Philos Trans R Soc Lond B Biol Sci 360:1935–1943. doi:10.1098/rstb.2005.1725. 16214751 PMC1609233

[50] Jones M, Ghoorah A, Blaxter M 2011 jMOTU and Taxonerator: turning DNA barcode sequences into annotated operational taxonomic units. PLoS One 6:e19259. doi:10.1371/journal.pone.0019259. 21541350 PMC3081837

[51] Overbeek R, Begley T, Butler RM, Choudhuri JV, Chuang HY, Cohoon M, de Crécy-Lagard V, Diaz N, Disz T, Edwards R, Fonstein M, Frank ED, Gerdes S, Glass EM, Goesmann A, Hanson A, Iwata-Reuyl D, Jensen R, Jamshidi N, Krause L, Kubal M, Larsen N, Linke B, McHardy AC, Meyer F, Neuweger H, Olsen G, Olson R, Osterman A, Portnoy V, Pusch GD, Rodionov DA, Rückert C, Steiner J, Stevens R, Thiele I, Vassieva O, Ye Y, Zagnitko O, Vonstein V 2005 The subsystems approach to genome annotation and its use in the project to annotate 1000 genomes. Nucleic Acids Res 33:5691–5702. doi:10.1093/nar/gki866. 16214803 PMC1251668

[52] Overbeek R, Olson R, Pusch GD, Olsen GJ, Davis JJ, Disz T, Edwards RA, Gerdes S, Parrello B, Shukla M, Vonstein V, Wattam AR, Xia F, Stevens R 2014 The SEED and the Rapid Annotation of microbial genomes using Subsystems Technology (RAST). Nucleic Acids Res 42:D206–D214. doi:10.1093/nar/gkt1226. 24293654 PMC3965101

[53] Ciccarelli FD, Doerks T, von Mering C, Creevey CJ, Snel B, Bork P 2006 Toward automatic reconstruction of a highly resolved tree of life. Science 311:1283–1287. doi:10.1126/science.1123061. 16513982

[54] Thompson JD, Higgins DG, Gibson TJ 1994 CLUSTAL W: improving the sensitivity of progressive multiple sequence alignment through sequence weighting, position-specific gap penalties and weight matrix choice. Nucleic Acids Res 22:4673–4680. doi:10.1093/nar/22.22.4673. 7984417 PMC308517

[55] Jones DT 2007 Improving the accuracy of transmembrane protein topology prediction using evolutionary information. Bioinformatics 23:538–544. doi:10.1093/bioinformatics/btl677. 17237066

[56] Krogh A, Larsson B, von Heijne G, Sonnhammer EL 2001 Predicting transmembrane protein topology with a hidden Markov model: application to complete genomes. J Mol Biol 305:567–580. doi:10.1006/jmbi.2000.4315. 11152613

[57] Duthie ES, Lorenz LL 1952 Staphylococcal coagulase: mode of action and antigenicity. J Gen Microbiol 6:95–107. doi:10.1099/00221287-6-1-2-95. 14927856

[58] Mayfield JA, Hammer ND, Kurker RC, Chen TK, Ojha S, Skaar EP, DuBois JL 2013 The chlorite dismutase (HemQ) from *Staphylococcus aureus* has a redox-sensitive heme and is associated with the small colony variant phenotype. J Biol Chem 288:23488–23504. doi:10.1074/jbc.M112.442335. 23737523 PMC5395028

[59] Wakeman CA, Hammer ND, Stauff DL, Attia AS, Anzaldi LL, Dikalov SI, Calcutt MW, Skaar EP 2012 Menaquinone biosynthesis potentiates haem toxicity in *Staphylococcus aureus*. Mol Microbiol 86:1376–1392. doi:10.1111/mmi.12063. 23043465 PMC3524387

[60] Stauff DL, Skaar EP 2009 *Bacillus anthracis* HssRS signalling to HrtAB regulates haem resistance during infection. Mol Microbiol 72:763–778. doi:10.1111/j.1365-2958.2009.06684.x. 19400785 PMC2891670

[61] Attia AS, Benson MA, Stauff DL, Torres VJ, Skaar EP 2010 Membrane damage elicits an immunomodulatory program in *Staphylococcus aureus*. PLoS Pathog 6:e1000802. doi:10.1371/journal.ppat.1000802. 20300601 PMC2837406

[62] Kreiswirth BN, Löfdahl S, Betley MJ, O’Reilly M, Schlievert PM, Bergdoll MS, Novick RP 1983 The toxic shock syndrome exotoxin structural gene is not detectably transmitted by a prophage. Nature 305:709–712. doi:10.1038/305709a0. 6226876

