# Supplementary figures and images for: Staphylococcus aureus HemX Modulates Glutamyl-tRNA Reductase Abundance To Regulate Heme Biosynthesis
Source: mBio. 2018 Feb 6;9(1):e02287-17. doi: 10.1128/mBio.02287-17 (PMC5801465; doi:10.1128/mBio.02287-17)

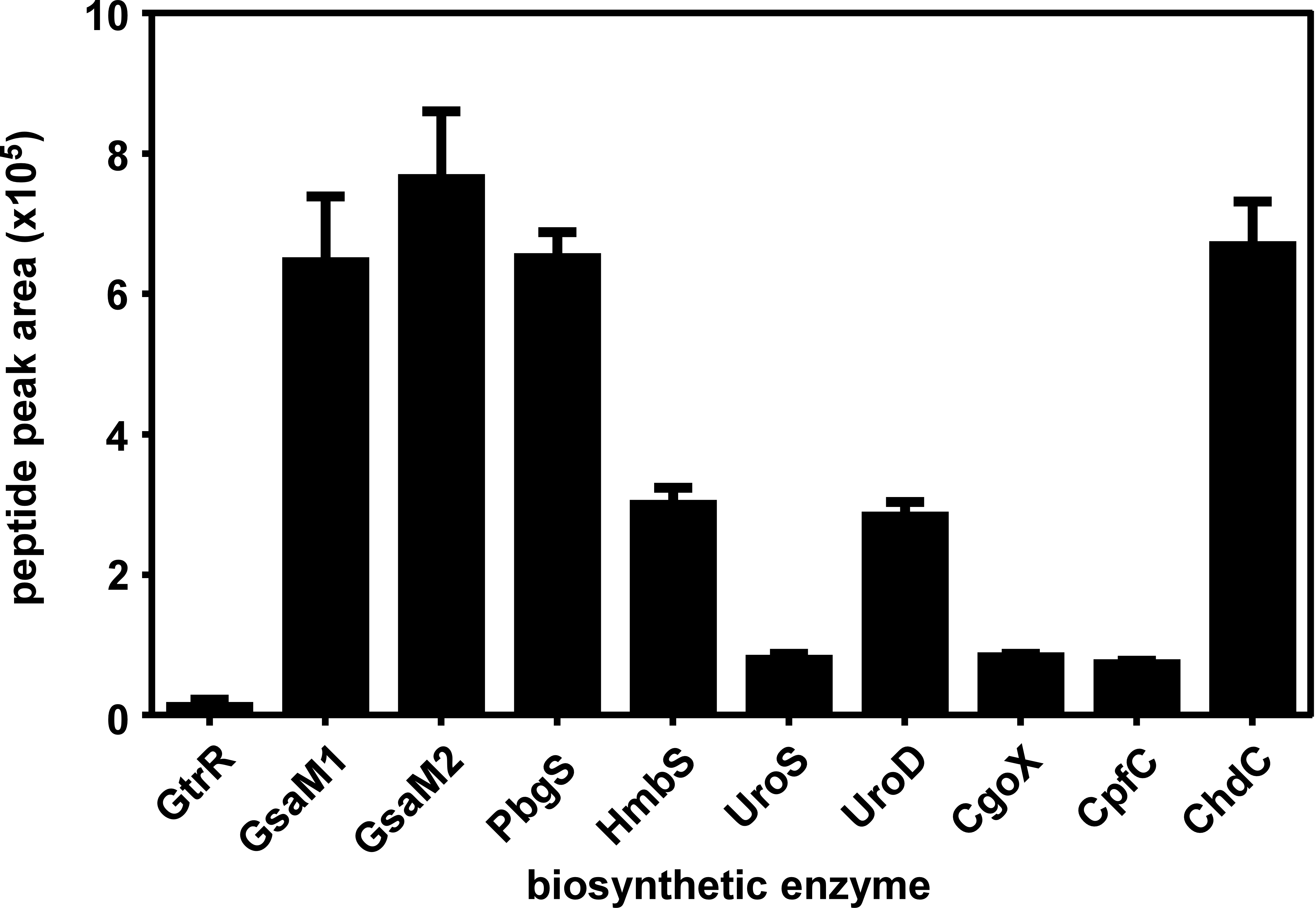

Supplement: FIG S1 [file mbo001183710sf1.tif]

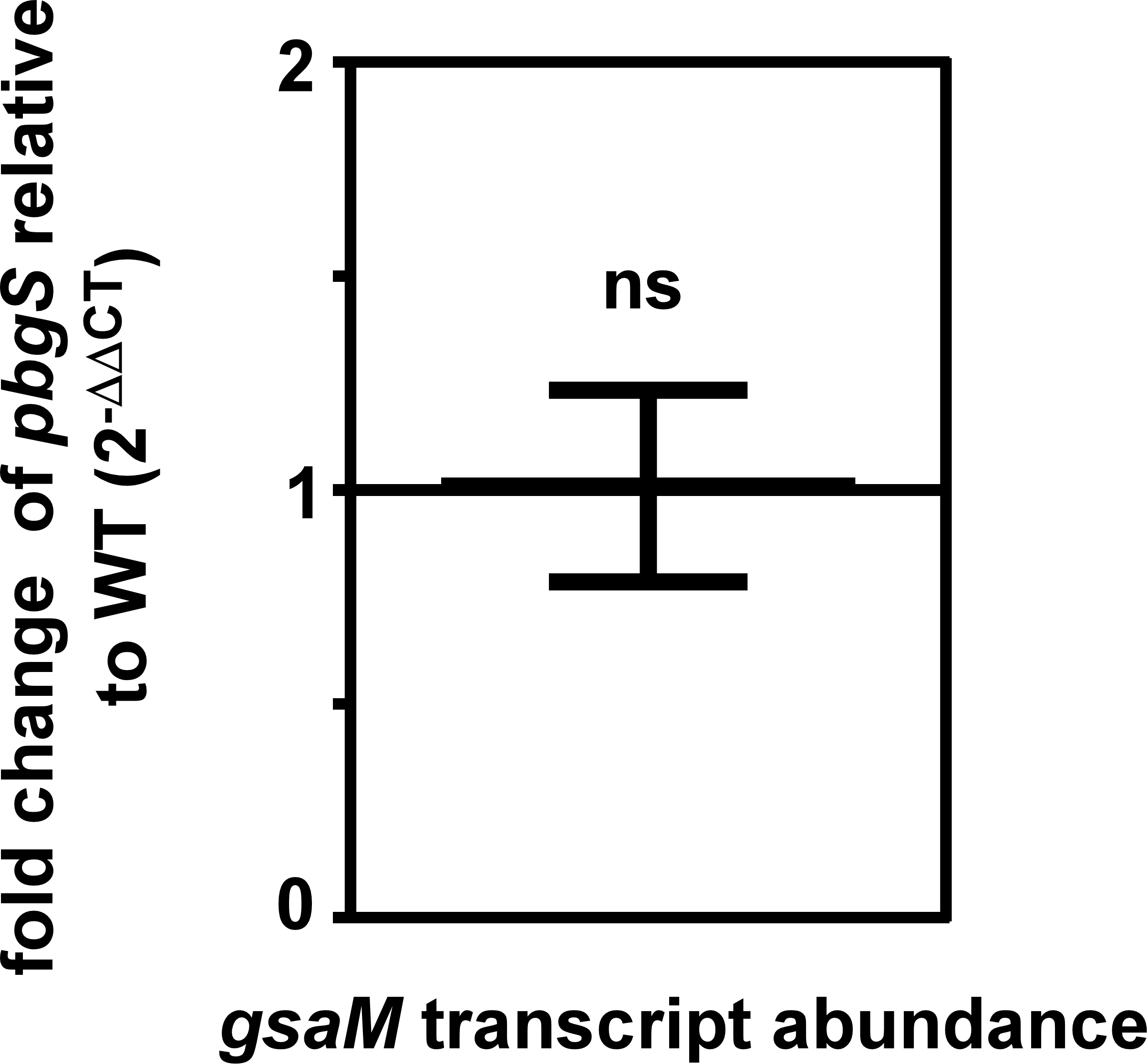

Supplement: FIG S2 [file mbo001183710sf2.tif]

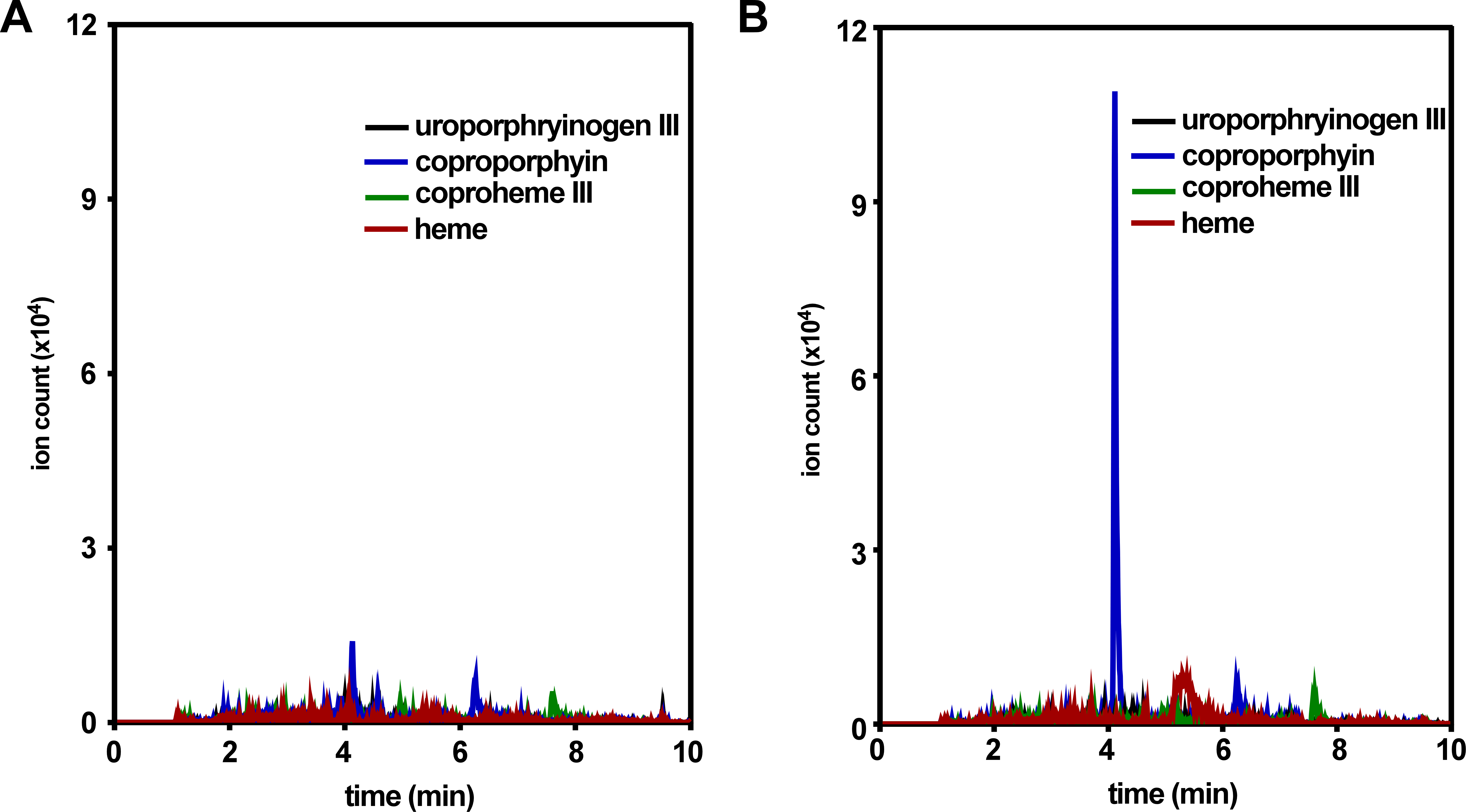

Supplement: FIG S3 [file mbo001183710sf3.tif]

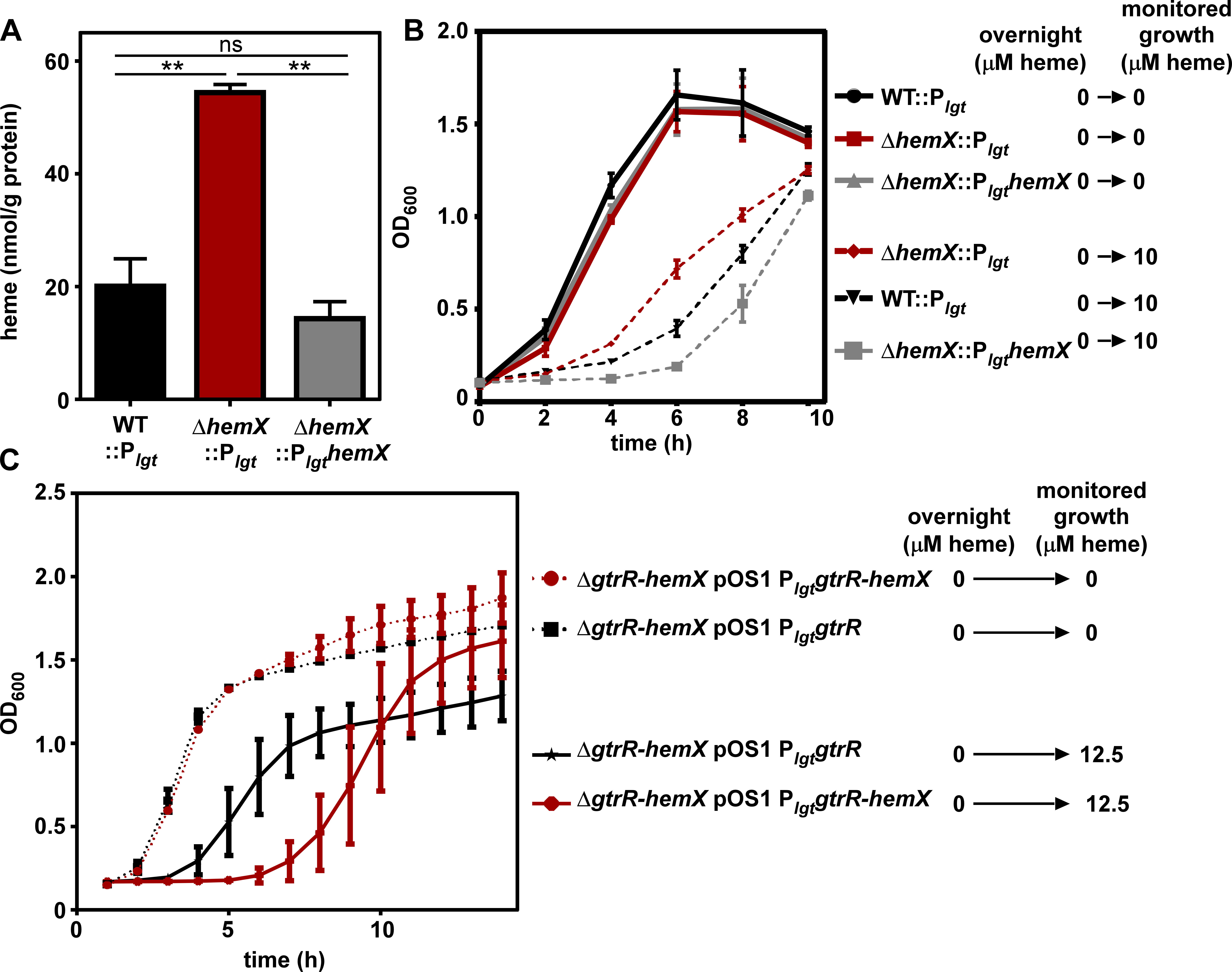

Supplement: FIG S4 [file mbo001183710sf4.tif]

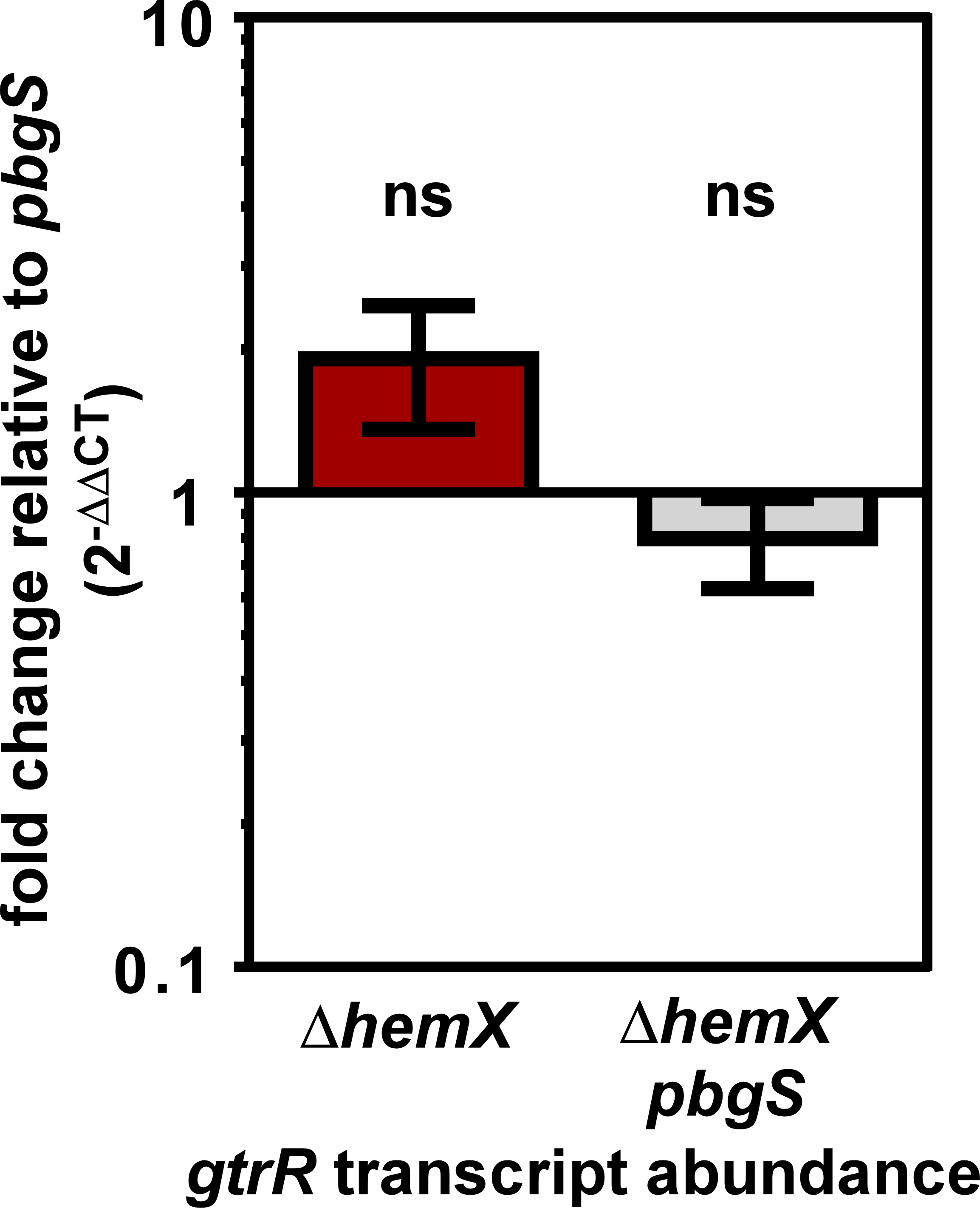

Supplement: FIG S5 [file mbo001183710sf5.tif]

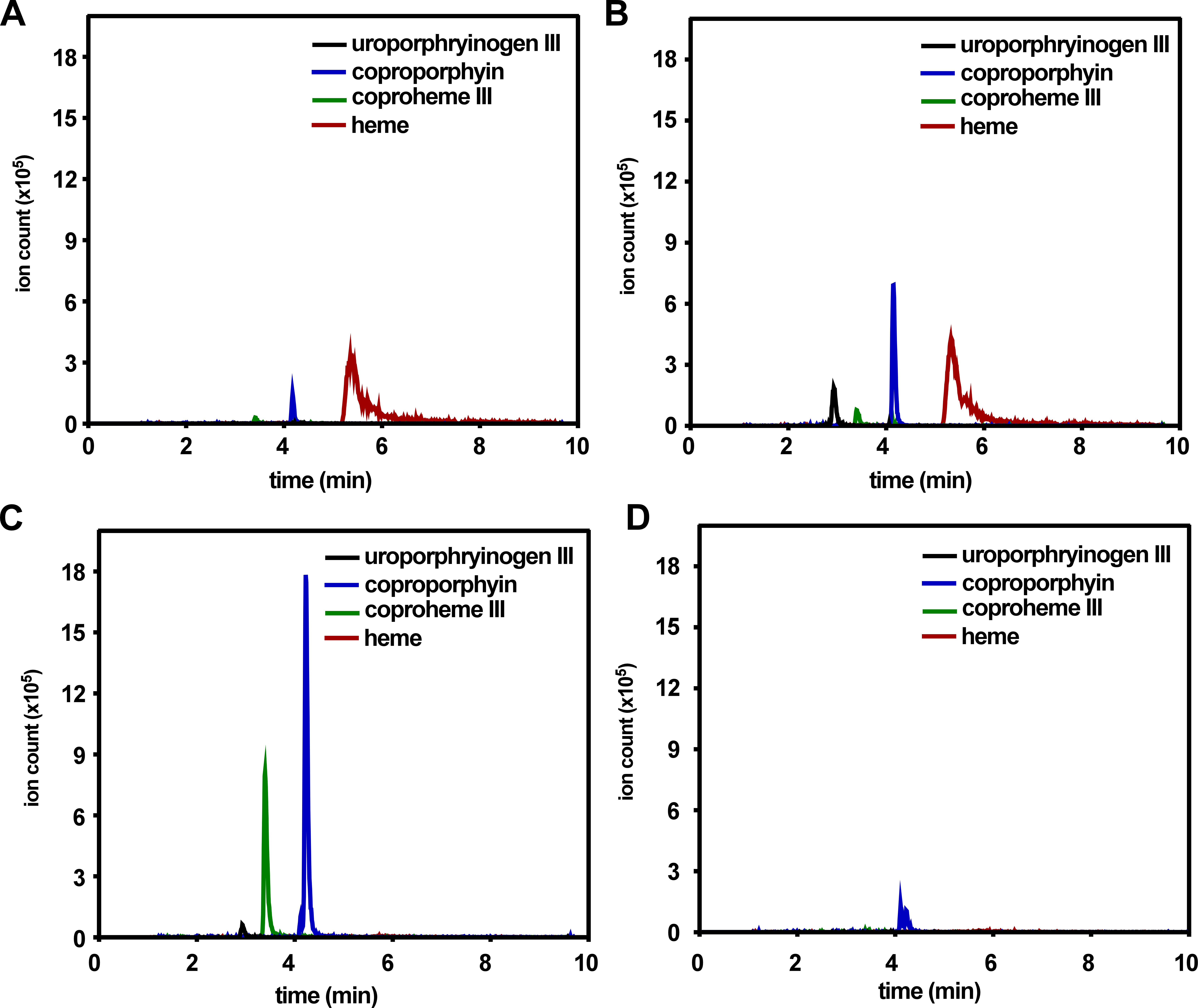

Supplement: FIG S6 [file mbo001183710sf6.tif]

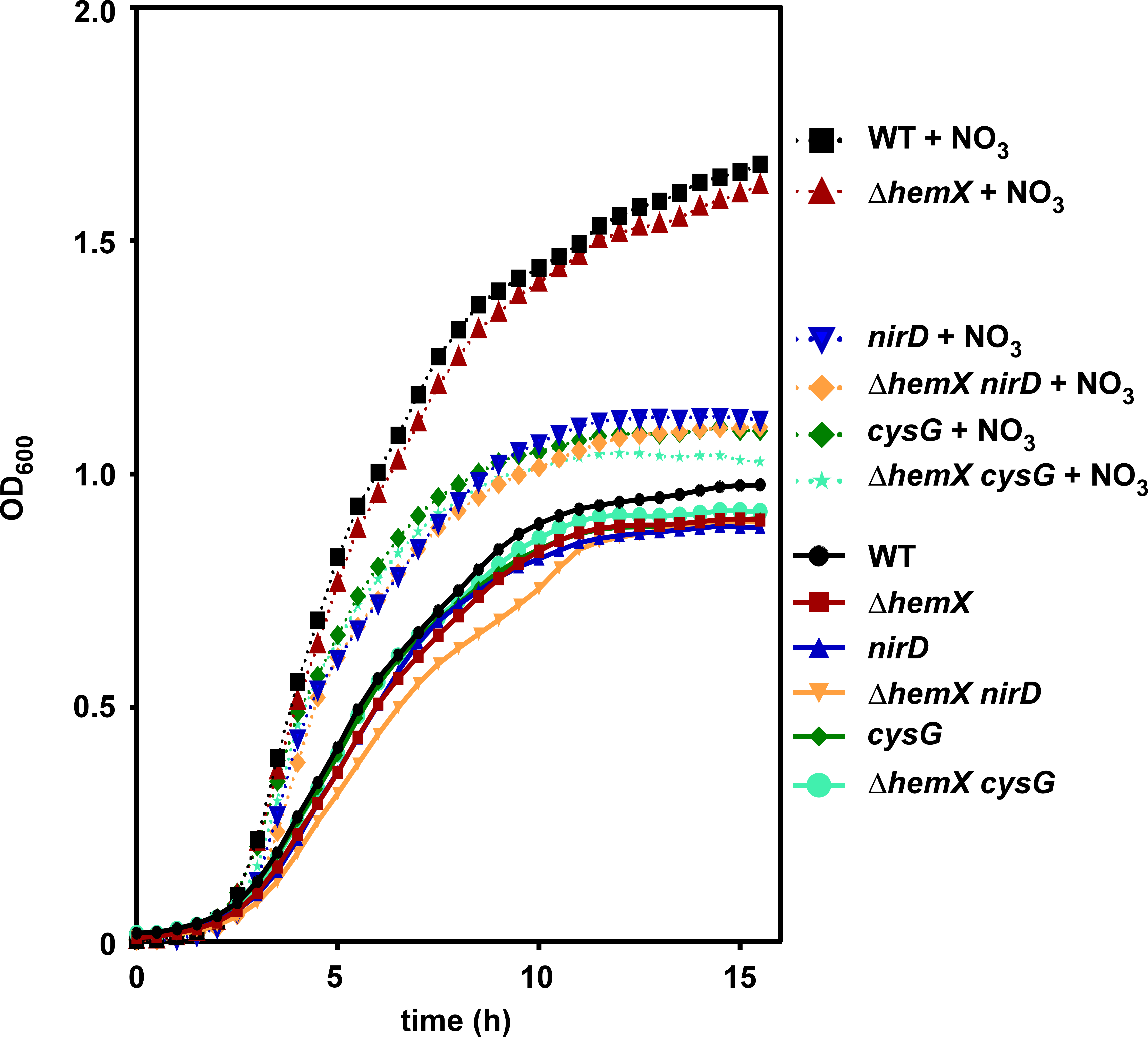

Supplement: FIG S7 [file mbo001183710sf7.tif]

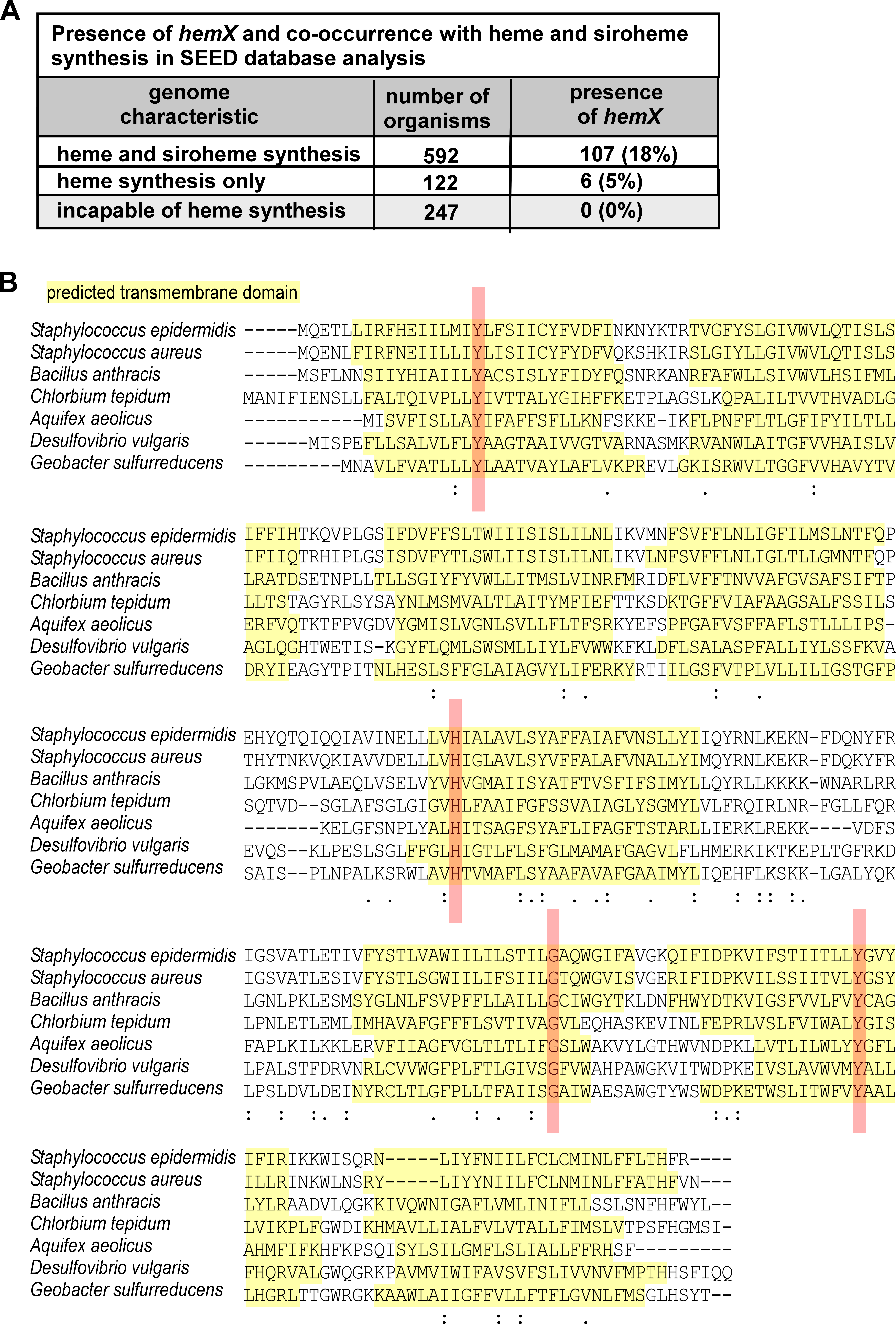

Supplement: FIG S8 [file mbo001183710sf8.tif]
