# Supplementary material for: Staphylococcus aureus HemX Modulates Glutamyl-tRNA Reductase Abundance To Regulate Heme Biosynthesis
Source: mBio. 2018 Feb 6;9(1):e02287-17. doi: 10.1128/mBio.02287-17 (PMC5801465; doi:10.1128/mBio.02287-17)
Supplement: TABLE S1 [file mbo001183710st1.docx]

**Supplemental Table 1. Plasmids and primers used in this study**

| **Plasmids** | | | |
| --- | --- | --- | --- |
| **Plasmid** | | **Description** | **Source** |
| pKOR1 | | Temperature sensitive allelic exchange vector | (1) |
| pKOR1Δ*hemX* | | Allelic exchange vector for deletion of *hemX* | This work |
| pXen1 (*luxABCDE*) | | Promoterless plasmid encoding *Photorhabdus luminescens* luciferase operon (*luxABCDE*), carbenicillin (*E. coli*) and chloramphenicol (*S. aureus*) selection | Xenogen; (6) |
| pXen1 P*_hrt_luxABCDE* | | *hrtAB* promoter cloned in pXen1 | (6) |
| pOS1 P*_hrt_xylE* | | *hrtAB* promoter driving *xylE* expression; carbenicillin (*E. coli*) and chloramphenicol (*S. aureus*) selection | (7) |
| pOS1 P*_isdA_gfp* | | *isdA* promoter driving *gfp* expression | This work |
| pOS1 P*_lgt_* | | *lgt* (constitutive) promoter | (8) |
| pOS1 P*_lgt_hemX* | | *hemX* cloned in pOS1 P*_lgt_* | This work |
| pJC1111 | | carbenicillin (*E. coli*) and cadmium chloride (*S. aureus*) selection | Torres lab; (9) |
| pJC1111 P*_lgt_* | |  | This work |
| pJC1111 P*_lgt_hemX* | |  | This work |
| pOS1 P*_lgt_gtrR* | | *gtrR* cloned in pOS1 P*_lgt_* | This work |
| pOS1 P*_lgt_gtrR-hemX* | | *gtrR-hemX* cloned in pOS1 P*_lgt_* | This work |
| **Primers** | | | |
| **Primer name** | **Sequence** | | |
| JC291 | GGGCCCGAGCTTAAGACT | | |
| JC292 | GATATCCCCTATAGTGAGTCGTATTAC | | |
| JC105 | TCACTATAGGGGATATCGCGTTTCAAATGCATTTTATTG | | |
| JC106 | TTATTTCTCATTATTCAAAACTAAAGATACGTCG | | |
| JC108 | GTTTTGAATAATGAGAAATAATCTAGAGCAATCC | | |
| JC109 | CTTAAGCTCGGGCCCTTTATAAAAGAATTTCACAGCATTTTTAG | | |
| JC103 | TCACTATAGGGGATATCTTGATGTGTTATAAAAGTGAAAAGC | | |
| JC104 | TTATTTCTCATTGAAACGCCCCCATATATAC | | |
| JC107 | GCGTTTCAATGAGAAATAATCTAGAGCAATCC | | |
| JC157 | GGGGGGCATATGCAAGAAAACCTGTTTATTCGATTCAATG | | |
| JC155 | GGGCGGATCCGGCTCAATTCACAAAATGTGTTGC | | |
| CG38 | ACGATCCGGGGAATTCCATATGTTATTTGTAGAGCTCATCCATGCCATG | | |
| CG39 | CTTGTTTGGATCCTCGAGGGATCCATGCCCGGGAGCAAAGGA | | |
| CG50 | GCCGAAGAATTCCAAAACATAATCCTCCTTTTTATG | | |
| CG51 | GCCAAGCATATGGTTGTTTTCCTCCTAAGGATACAA | | |
| JC101 | GCGGCATATGGCCATGCATTTTATTGCAATTAGTAT | | |
| JC102 | GCGGGGATCCGCCTTATTCAAAACTAAAGATACGTC | | |
| JC83 | GTGATTTTAGTCATACGCGCTTC | | |
| JC84 | GAACCCTGATATTTCAATTGCTG | | |
| JC53 | TTTGTTCCGAAATTGTTGCA | | |
| JC54 | GAAAGATCCTATTAAACAGGCCAAA | | |
| JC55 | AACCAATCATTGAACCTGCTC | | |
| JC56 | CATTTGATACGGCTTTAAGTCGT | | |
| JC81 | CAGTCTTACCTGCTCGATTCC | | |
| JC82 | GTGGAATATTCGTTGCCATACC | | |
| HS1 | TGTTGGGTGTTGTTCCAGTG | | |
| HS2 | GCTTAACCCTGGCAATTTACG | | |
| HS3 | CGAGAAATGGCAAAAGAAGG | | |
| HS4 | TTCTTTCGTGTGTGCCGTAG | | |
| JC158 | GCCGTCGACGCCACTAATGATTTATTATGTAGTGGTTC | | |
| JC229 | GGGGGAAACACTACCCCCTTG | | |
| JC184 | GATATGGACCCAGACGATACAATTG | | |
| JC185 | GCACCACGACGTAATGAACTAG | | |
