# Supplementary material for: Staphylococcus aureus HemX Modulates Glutamyl-tRNA Reductase Abundance To Regulate Heme Biosynthesis
Source: mBio. 2018 Feb 6;9(1):e02287-17. doi: 10.1128/mBio.02287-17 (PMC5801465; doi:10.1128/mBio.02287-17)
Supplement: TEXT S1 [file mbo001183710s1.docx]

**SUPPLEMENTAL MATERIAL**

**SUPPLEMENTAL MATERIALS AND METHODS**

**Bacterial strains and reagents**

*Deletion of hemX*. Deletion of *hemX* by allelic exchange was performed as described in (1) with some modifications. The pKOR1 backbone was amplified by PCR using JC291/292. The ~1kb flanking regions were amplified from *S. aureus* Newman genomic DNA using JC105/106 (upstream flanking) and JC108/109 (downstream flanking). The allelic exchange protocol to create an in-frame unmarked deletion of *hemX* was performed in *S. aureus* WT, *pbgS*, and Δ*chdC*. Deletion of *hemX* was confirmed by PCR using JC184/185.

*Deletion of gtrR-hemX*. Deletion of *gtrR*-*hemX,* which are co-transcribed from adjacent positions in the chromosome, by allelic exchange was performed as described above. The ~1kb flanking regions were amplified from *S. aureus* Newman genomic DNA using JC103/104 (upstream flanking) and JC107/109 (downstream flanking). The allelic exchange protocol to create an in-frame unmarked deletion of *gtrR-hemX* was performed in *S. aureus* WT. Deletion was confirmed by PCR using JC184/185.

*pOS1 P_isdA_-gfp cloning*. The sequence for codon optimized *gfp* was amplified from pGFP-F (2) using primers CG38/39 and subsequently assembled using Gibson Assembly into pOS1 that was digested with NdeI and BamHI. P*_isdA_* was amplified from *S. aureus* Newman genomic DNA using primers CG50/51 and was ligated into pOS1 *gfp* after digestion with EcoRI and NdeI to produce pOS1 P­*_isdA­_gfp*.

*pOS1 P_lgt_gtrR and P_lgt_gtrR-hemX cloning. gtrR* was amplified from *S. aureus* Newman genomic DNA using primers JC101/102, while *gtrR-hemX* was amplified using JC101/155 and ligated into pOS1 P*_lgt_* after digestion of PCR products and vector with NdeI and BamHI to produce pOS1 P*_lgt_gtrR* and pOS1 P*_lgt_gtrR-hemX*

**LC-qTOF-MS porphyrin quantification**

*S. aureus* strains were streaked to TSA and grown for 18 h at 37°C. For WT and Δ*hemX*, single colonies were used to start 5 mL cultures in TSB and grown for 10 h at 37°C. One hundred µL of this culture was inoculated into 50 mL of TSB in a 250 mL Erlenmeyer flask and grown at 37°C for 14 h. For Δ*chdC* and Δ*hemX* Δ*chdC*, multiple colonies colonies were used to start 5 mL cultures in TSB containing 2 µM heme and grown for 10 h at 37°C. Four hundred µL of this culture was inoculated into 200 mL of TSB in a 500 mL Erlenmeyer flask and grown at 37°C for 14 h. Total CFU were determined by serial dilution and plating to TSA for enumeration, and cells were collected by centrifugation and frozen at -80°C. Any negligible levels of heme measured in Δ*chdC* or Δ*hemX* Δ*chdC* is likely carry-over from the heme containing initial culture.

*Porphyrin extraction.* To the cell pellets, 1 mL of 1M HCl:DMSO (1:1, v/v) was added and samples vortexed, sonicated (2x2 min total, 1 sec on/1 sec off, ice, Ultrasonic Homogenizer, Biologistics Inc. Model 3000), and centrifuged (10,000 rpm, 5 min, 4°C). Supernatants were collected and kept in the dark. Pellets were resuspended in 1 mL of 1M HCl:DMSO (1:1, v/v), vortexed vigorously for 30 s twice, and centrifuged again as above. Supernatants were pooled and the resuspension/centrifugation cycle repeated. The pooled supernatants were filtered using a 0.22 µM PES syringe filter (GE Healthcare Life Sciences) and subsequently diluted to 25 mL with ddH_2_O. The extracts were purified using a Sep-Pak® Vac 3cc tC_18_ cartridge (Waters 036815), eluted with 2 mL of acetonitrile + 0.1% TFA then 2 mL of methanol. Extracted porphyrins were concentrated under N_2_(g) purge and resuspended in 50 µL of acetonitrile + 0.1% TFA. All samples were immediately dispensed into vials for analysis. Excess remaining samples were stored at -20°C in the dark.

*Preparation of standards*. 2 mM stock solutions of porphyrin standards (porphobilinogen, uroporphyrin III, coproporphyrin I and III, coproheme III, protoporphyrin IX, and heme *b*) were individually prepared in DMSO and then mixed to make a 100 µM (final concentration of each porphyrin). This stock was then diluted to 0.25-6 µM in acetonitrile + 0.1% TFA.

*LC-qTOF-MS analysis*. Twenty-five µL samples were diluted with 75 µL of ultrapure water immediately prior to measurement. The column (Agilent PLRP-S 100Å, 4.6x150x5 µm) was equilibrated to an 85:15 ratio of solvent A (ultrapure water with 0.1% formic acid) to solvent B (acetonitrile + 0.1% formic acid). LC separations were achieved by linear gradient elution, transitioning from 15% to 95% solvent B over 6 min followed by a 2 min hold at 95% B. The column was re-equilibrated to 15% solvent B for 2 min between injections of the same sample (two technical replicates per sample). Two blank runs were implemented between samples (2 µL injection volume, 600 µL/min flow rate, 50°C) to ensure against column holdover of analytes. Electrospray ionization mass spectrometry analysis was carried out in positive mode with a capillary voltage of 2 Hz (Agilent 6538 UHD q-TOF).

Data were analyzed using MassHunter Qualitative Analysis Software and MZmine 2. Values for *m/z* were determined empirically for standards. From the total ion chromatogram (TIC) traces, extracted ion chromatograms (EICs) were derived for each individual standard on the basis of its mass per charge (*m/z*) in positive ion mode, which is equivalent to the exact mass of its positive ion (parent compound plus H^+^). Peaks associated with each analyte were integrated and areas were plotted versus concentration (µM). Linear regression analysis (Kaleidagraph) was used to determine the correlation coefficient between integrated peak area and porphyrin concentration (slope of standard curve, m_porph_).

All cell samples were analyzed for the full set of standards. Only analytes present above the limit of detection are reported; detection limits were at least 250 nM per injection; for a saturated culture with 10^9^ CFU mL-1, this is equivalent to 250 x 10^-9^ pmoles CFU^-1^). For the quantification of analytes from cells, values for the integrated peak intensities (measured in units of *ion counts*) were converted to units of concentration (µmol/L injected) via: counts x (m_porph_)^-1^. The concentration of each analyte in the injected volumes [A] was subsequently converted to units of nmol analyte per CFU in sample as: [A] x (volume used to resuspend dried sample) x (CFUs in analyzed cell pellet)^-1^. Reported values are averages of technical replicates.

**LC-MRM-MS/MS**

Cells were collected by centrifugation, washed in PBS, and resuspended in 500 µl of TSM (100 mM Tris, pH 7; 500 mM sucrose; 10 mM MgCl_2_) containing 4 µg of lysostaphin. Cells were incubated for 60 min at 37° C. Protoplasts were collected by centrifugation and resuspended in 150 µl of PBS containing 100 µM PMSF. Protoplasts were lysed by sonication and the soluble lysate was collected after centrifugation to remove unlysed protoplasts. Protein content was quantified using the Pierce BCA Assay (Thermo) and lysate was added to 4X NuPAGE LDS sample buffer with reducing agent (Thermo) and 100 µg of total protein for each replicate was subject to brief SDS-PAGE in NuPAGE gel (Thermo) according to manufacturer’s instructions.

The gel regions were excised and subjected to in-gel trypsin digestion and peptide extraction as previously described (3). Proteins were quantified with multiple reaction monitoring (4). Representative peptides for each protein were selected based on theoretical trypsin digest. Skyline software (University of Washington, MacCoss lab (5)) was used to set up scheduled, targeted MRM methods and three to five MS/MS transitions were monitored per peptide.

The 20 μL digest was transferred to an autosampler vial and 4 μL sample per run was injected via autosampler (NanoAcuity HPLC system, Waters) onto a vented column setup utilizing a 40 mm by 0.1 mm (Jupiter 5 micron, 300A) kasil fritted trap followed by a 250 mm by 0.1 mm (Jupiter 3 micron, 300A), self-packed analytical column coupled directly to a TSQ-Vantage (Thermo Scientific) via a nanoelectrospray source. After trapping and equilibration, peptides were resolved using a 90-minute aqueous to organic gradient (solvent A = 0.1% FA in water and B = 0.1% FA in ACN) operating at 400 nL/min. A series of unscheduled runs determined retention times and the most useful transitions to monitor and then a scheduled instrument method encompassing a 8-minute window around the measured retention time along with calculated collision energies was created using Skyline. Q1 peak width resolution was set to 0.7, collision gas pressure was 1 mTorr, and utilized an EZ method cycle time of 3 seconds. The resulting RAW instrument files were imported into Skyline for peak-picking and quantitation. Transition or fragment ion peak areas were summed to represent the intensity of endogenous peptides, and normalized to *S. aureus* Newman GyrA as noted.

**Supplemental References**

1. **Bae T, Schneewind O.** 2006. Allelic replacement in *Staphylococcus aureus* with inducible counter-selection. Plasmid **55:**58-63.

2. **Bose JL, Fey PD, Bayles KW.** 2013. Genetic tools to enhance the study of gene function and regulation in *Staphylococcus aureus*. Applied and environmental microbiology **79:**2218-2224.

3. **Shevchenko A, Wilm M, Vorm O, Mann M.** 1996. Mass spectrometric sequencing of proteins silver-stained polyacrylamide gels. Anal Chem **68:**850-858.

4. **Gerber SA, Rush J, Stemman O, Kirschner MW, Gygi SP.** 2003. Absolute quantification of proteins and phosphoproteins from cell lysates by tandem MS. Proc Natl Acad Sci U S A **100:**6940-6945.

5. **MacLean B, Tomazela DM, Shulman N, Chambers M, Finney GL, Frewen B, Kern R, Tabb DL, Liebler DC, MacCoss MJ.** 2010. Skyline: an open source document editor for creating and analyzing targeted proteomics experiments. Bioinformatics **26:**966-968.

6. **Mike LA, Dutter BF, Stauff DL, Moore JL, Vitko NP, Aranmolate O, Kehl-Fie TE, Sullivan S, Reid PR, DuBois JL, Richardson AR, Caprioli RM, Sulikowski GA, Skaar EP.** 2013. Activation of heme biosynthesis by a small molecule that is toxic to fermenting *Staphylococcus aureus*. Proc. Natl. Acad. Sci. USA **110:**9206-8211.

7. **Torres VJ, Stauff DL, Pishchany G, Bezbradica JS, Gordy LE, Iturregui J, Anderson KL, Dunman PM, Joyce S, Skaar EP.** 2007. A *Staphylococcus aureus* regulatory system that responds to host heme and modulates virulence. Cell Host Microbe **1:**109-119.

8. **Schneewind O, Model P, Fischetti VA.** 1992. Sorting of protein A to the staphylococcal cell wall. Cell **70:**267-281.

9. **Chen J, Yoong P, Ram G, Torres VJ, Novick RP.** 2014. Single-copy vectors for integration at the SaPI1 attachment site for *Staphylococcus aureus*. Plasmid **76C:**1-7.
